# Supplementary material for: Caveolin-1 dolines form a distinct and rapid caveolae-independent mechanoadaptation system
Source: Nat Cell Biol. 2022 Dec 21;25(1):120–33. doi: 10.1038/s41556-022-01034-3 (PMC9859760; doi:10.1038/s41556-022-01034-3)
Supplement: Supplementary file 1 — This pdf file includes Supplementary Figs. 1–3, the corresponding supplementary figure legends and Supplementary Note 1. [file 41556_2022_1034_MOESM1_ESM.pdf]

nature cell biology

Article https://doi.org/10.1038/s41556-022-01034-3

Caveolin-1 dolines form a distinct and rapid caveolae-independent mechanoadaptation system

In the format provided by the
authors and unedited

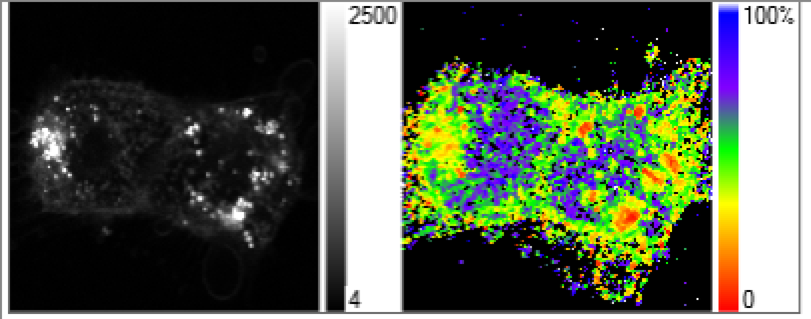

Cell1 w8p2 PTRFko NB-25 ex900 0_29mW z-4 ch3 [Freq1]
☑ Highlight Image Data: S1->S2 Fraction; Color: Palette
Image Size: 256 x 256

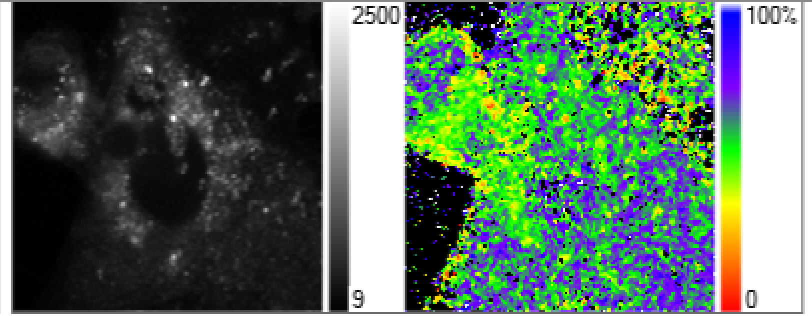

Cell2 w6p2 PTRFko NB-25 ex900 0_29mW ch3 [Freq1]
☑ Highlight Image Data: S1->S2 Fraction; Color: Palette
Image Size: 256 x 256

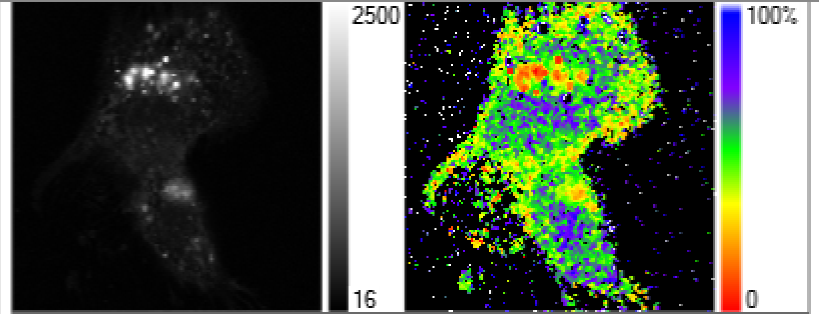

Cell3 w7p2 PTRFko NB-25 ex900 0_29mW z-1 ch3 [Freq1]
☑ Highlight Image Data: S1->S2 Fraction; Color: Palette
Image Size: 256 x 256

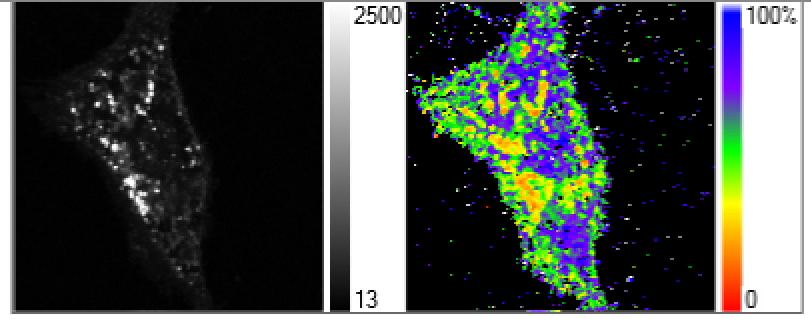

Cell4 w8p2 PTRFko NB-25 ex900 0_29mW ch3 [Freq1]
☑ Highlight Image Data: S1->S2 Fraction; Color: Palette
Image Size: 256 x 256

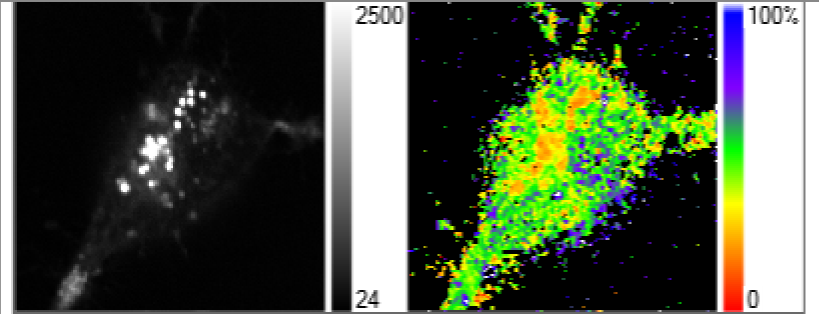

Cell2 w7p2 PTRFko NB-25 ex900 0_29mW ch3 [Freq1]
☑ Highlight Image Data: S1->S2 Fraction; Color: Palette
Image Size: 256 x 256

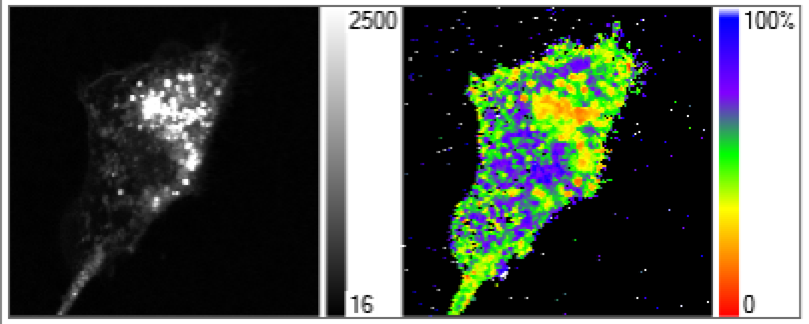

Cell2 w8p2 PTRFko NB-25 ex900 0_29mW ch3 [Freq1]
☑ Highlight Image Data: S1->S2 Fraction; Color: Palette
Image Size: 256 x 256

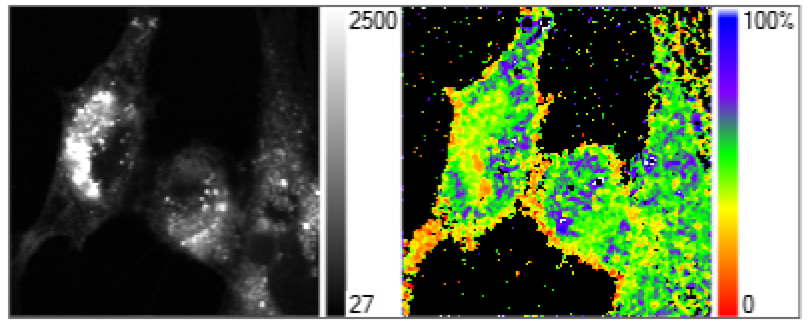

Cell3 w6p2 PTRFko NB-25 ex900 0_29mW ch3 [Freq1]
☑ Highlight Image Data: S1->S2 Fraction; Color: Palette
Image Size: 256 x 256

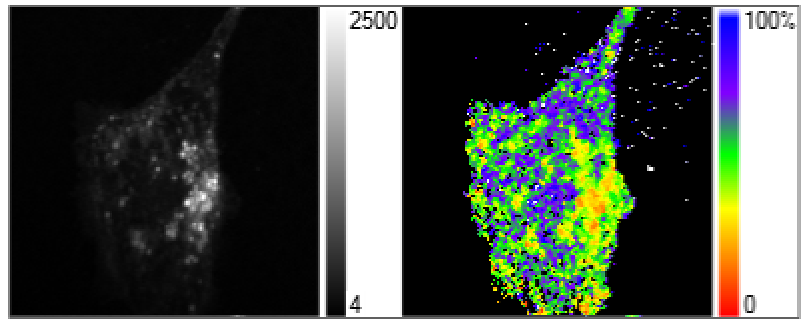

Cell3 w8p2 PTRFko NB-25 ex900 0_29mW ch3 [Freq1]
☑ Highlight Image Data: S1->S2 Fraction; Color: Palette
Image Size: 256 x 256

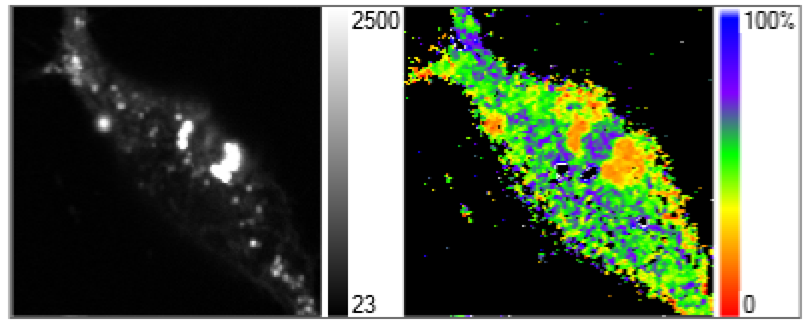

Cell6 w7p2 PTRFko NB-25 ex900 0_29mW ch3 [Freq1]
☑ Highlight Image Data: S1->S2 Fraction; Color: Palette
Image Size: 256 x 256

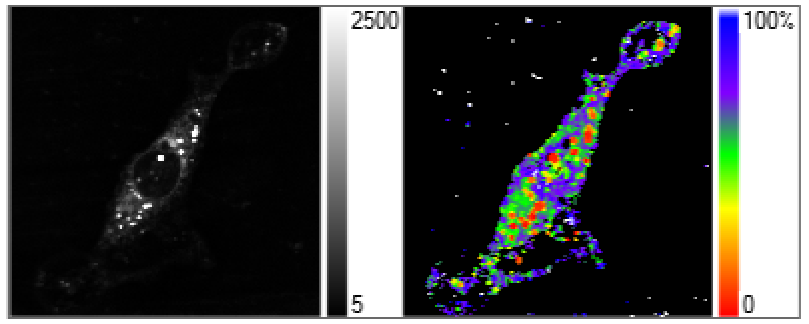

11 w1 PTRF-ko z10 50p [Freq1]
☑ Highlight Image Data: S1->S2 Fraction; Color: Palette
Image Size: 256 x 256

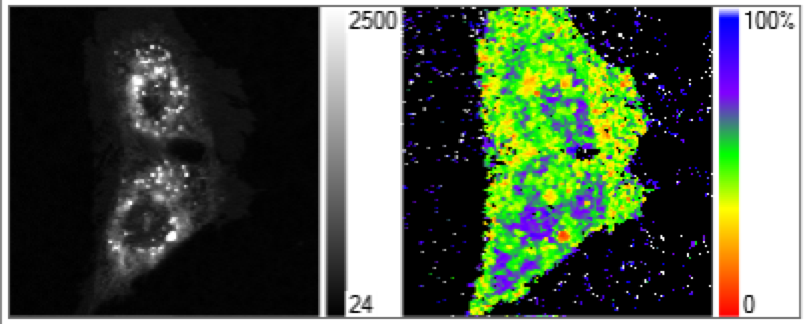

Cell1 w8p1 PTRFko NB-25 ex900 0_29mW ch3 [Freq1]
☑ Highlight Image Data: S1->S2 Fraction; Color: Palette
Image Size: 256 x 256

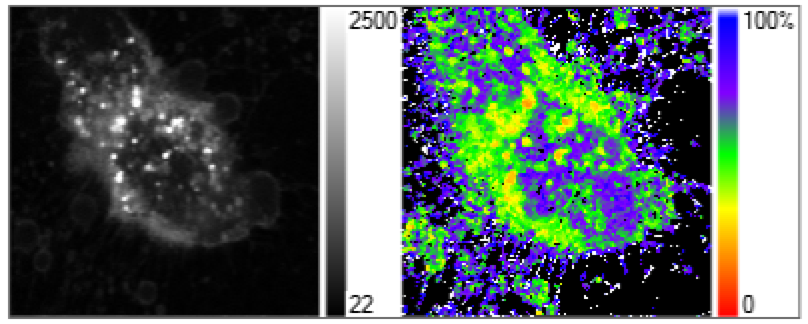

Cell2 w8p1 PTRFko NB-25 ex900 0_29mW z1 ch3 [Freq1]
☑ Highlight Image Data: S1->S2 Fraction; Color: Palette
Image Size: 256 x 256

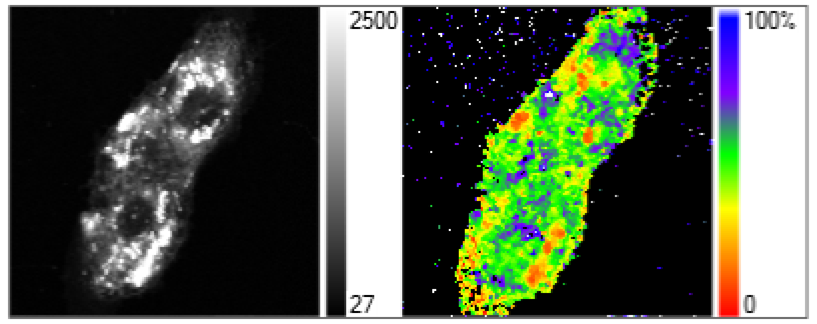

Cell5 w6p2 PTRFko NB-25 ex900 0_29mW ch3 [Freq1]
☑ Highlight Image Data: S1->S2 Fraction; Color: Palette
Image Size: 256 x 256

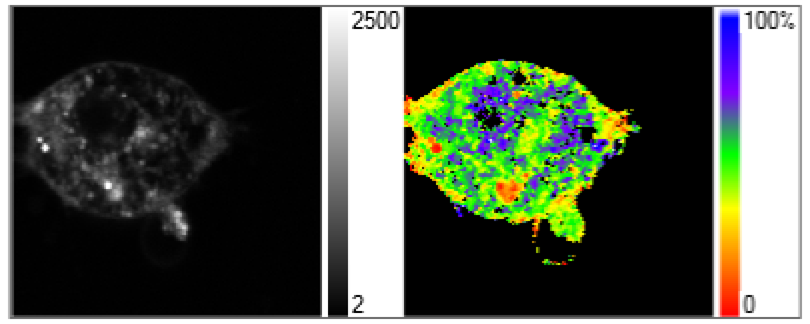

Cell5 w7p2 PTRFko NB-25 ex900 0_29mW ch3 [Freq1]
☑ Highlight Image Data: S1->S2 Fraction; Color: Palette
Image Size: 256 x 256

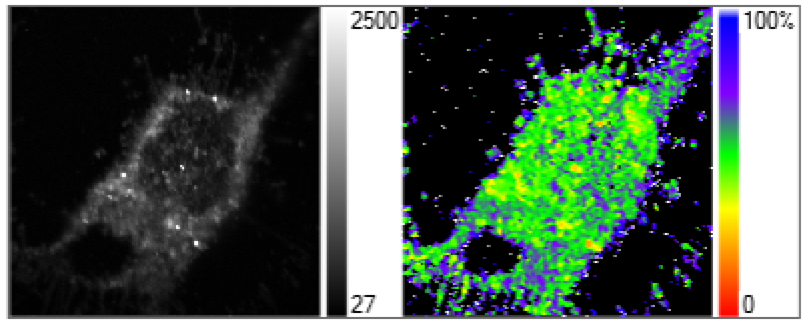

Cell1 w8p1 PTRFko NB-25 ex900 0_24mW ch3 [Freq1]
☑ Highlight Image Data: S1->S2 Fraction; Color: Palette
Image Size: 256 x 256

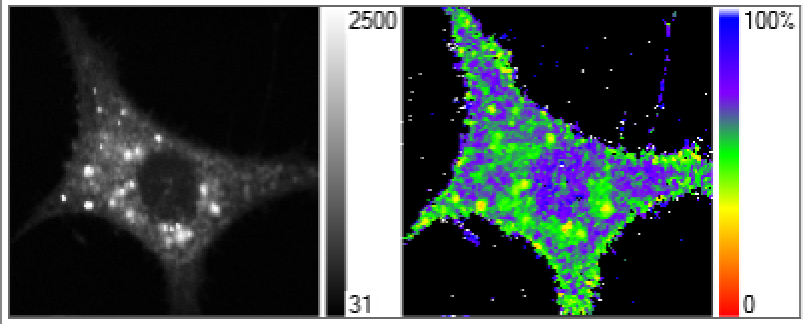

Cell1 w7p1 PTRFko NB-25 ex900 0_24mW ch3 [Freq1]
☑ Highlight Image Data: S1->S2 Fraction; Color: Palette
Image Size: 256 x 256

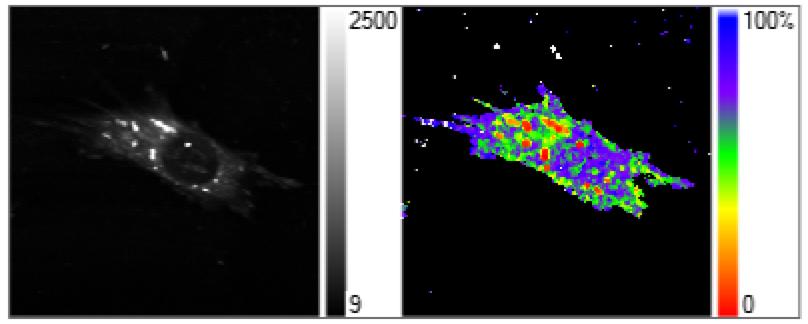

3 w1 PTRF-ko z00 50p [Freq1]
☑ Highlight Image Data: S1->S2 Fraction; Color: Palette
Image Size: 256 x 256

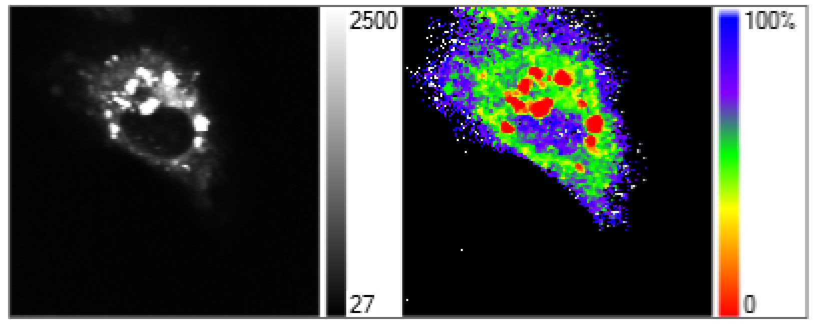

PTRF KO 25 NBD Cholesterol test_2 [Freq1]
☑ Highlight Image Data: S1->S2 Fraction; Color: Palette
Image Size: 256 x 256

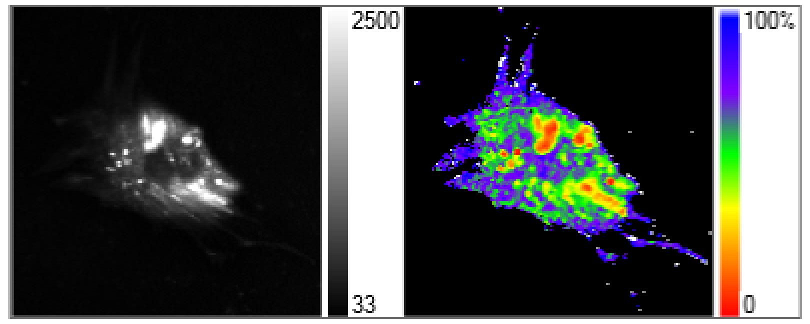

6 w1 PTRF-ko z00 60p [Freq1]
☑ Highlight Image Data: S1->S2 Fraction; Color: Palette
Image Size: 256 x 256

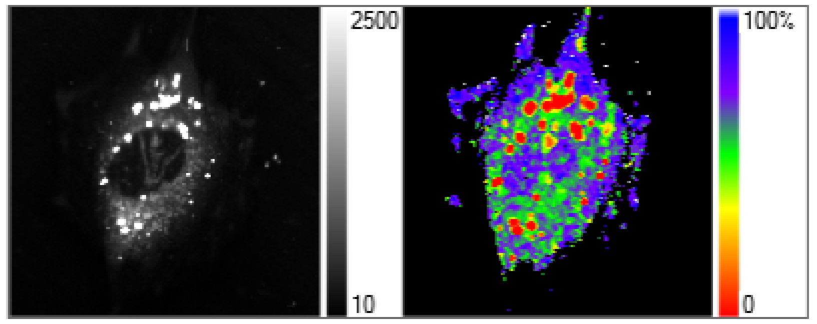

7 w1 PTRF-ko z00 50p [Freq1]
☑ Highlight Image Data: S1->S2 Fraction; Color: Palette
Image Size: 256 x 256

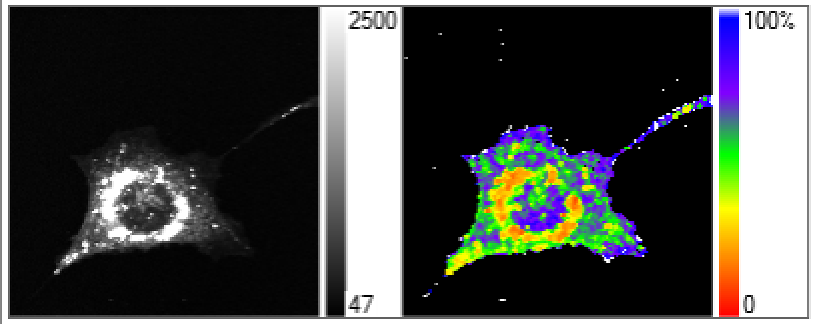

PTRF KO 25 NBD Cholesterol test_3 [Freq1]
☑ Highlight Image Data: S1->S2 Fraction; Color: Palette
Image Size: 256 x 256

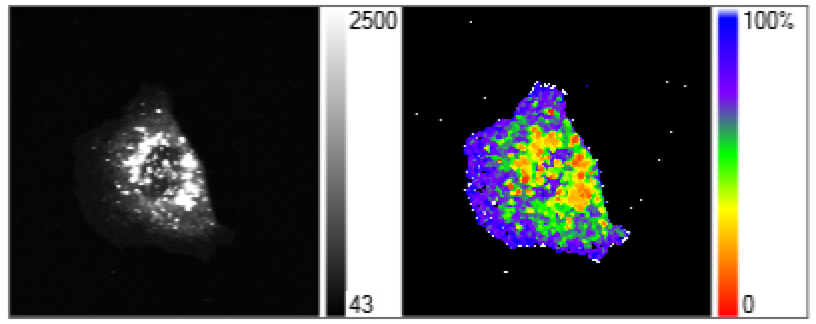

PTRF KO 25 NBD Cholesterol test_5 [Freq1]
☑ Highlight Image Data: S1->S2 Fraction; Color: Palette
Image Size: 256 x 256

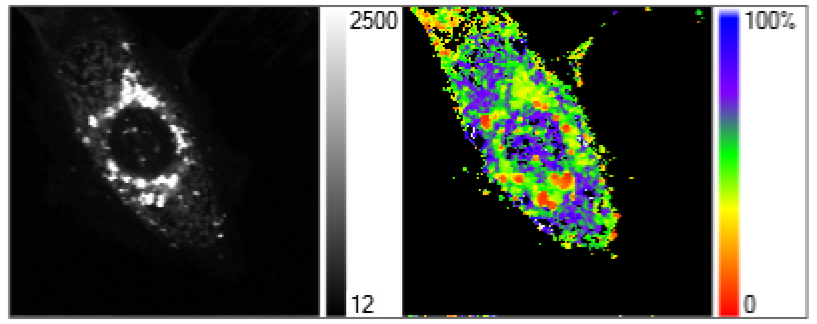

Cell4 w6p2 PTRFko NB-25 ex900 0_29mW z1 ch3 [Freq1]
☑ Highlight Image Data: S1->S2 Fraction; Color: Palette
Image Size: 256 x 256

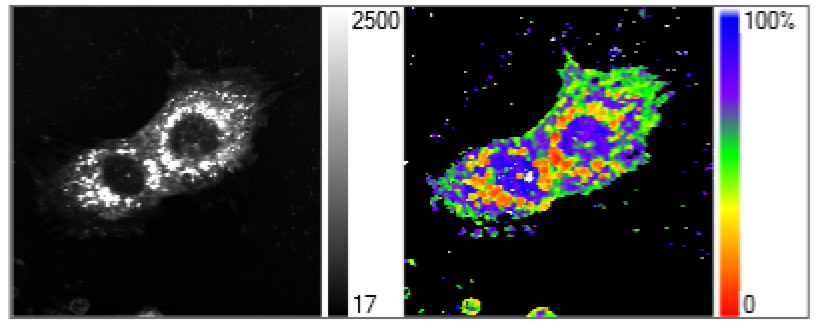

3 w1 PTRF-ko z01 50p [Freq1]
☑ Highlight Image Data: S1->S2 Fraction; Color: Palette
Image Size: 256 x 256

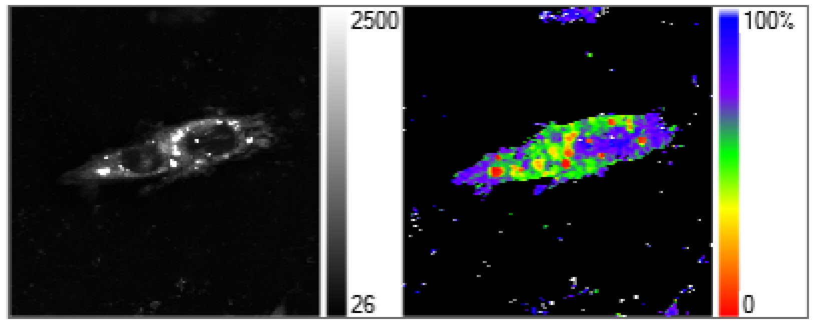

8 w1 PTRF-ko z00 45p [Freq1]
☑ Highlight Image Data: S1->S2 Fraction; Color: Palette
Image Size: 256 x 256

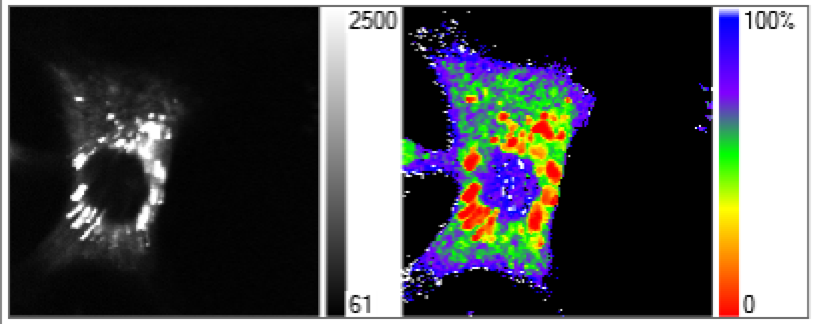

PTRF KO 25 NBD Cholesterol test_4 [Freq1]
☑ Highlight Image Data: S1->S2 Fraction; Color: Palette
Image Size: 256 x 256

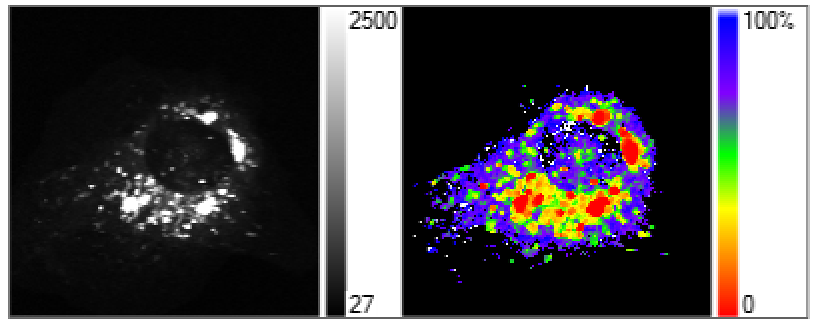

PTRF KO 25 NBD Cholesterol test_4 [Freq1]
☑ Highlight Image Data: S1->S2 Fraction; Color: Palette
Image Size: 256 x 256

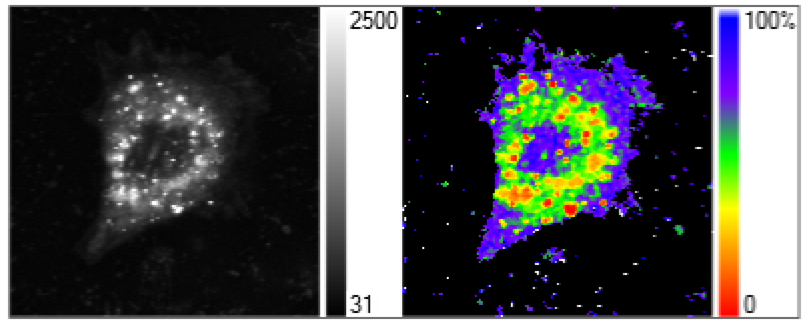

7 w1 PTRF-ko z01 45p [Freq1]
☑ Highlight Image Data: S1->S2 Fraction; Color: Palette
Image Size: 256 x 256

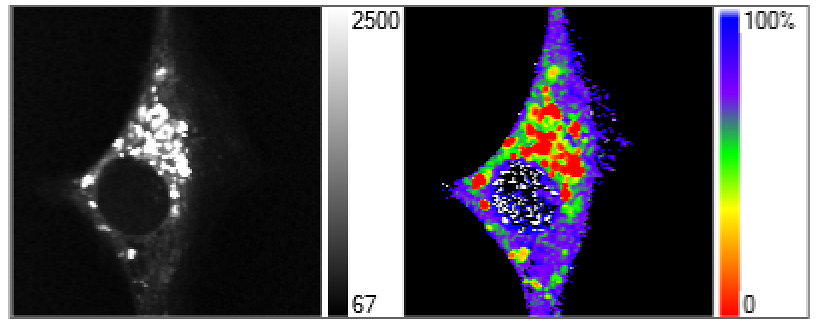

PTRF KO 25 NBD Cholesterol test_5 [Freq1]
☑ Highlight Image Data: S1->S2 Fraction; Color: Palette
Image Size: 256 x 256

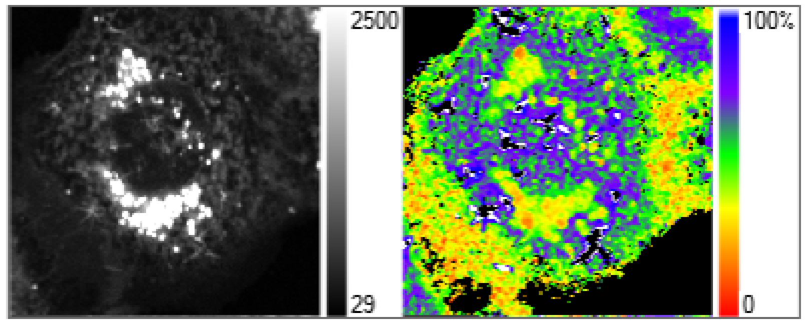

Cell6 w6p2 PTRFko NB-25 ex900 0_29mW z1 ch3 [Freq1]
☑ Highlight Image Data: S1->S2 Fraction; Color: Palette
Image Size: 256 x 256

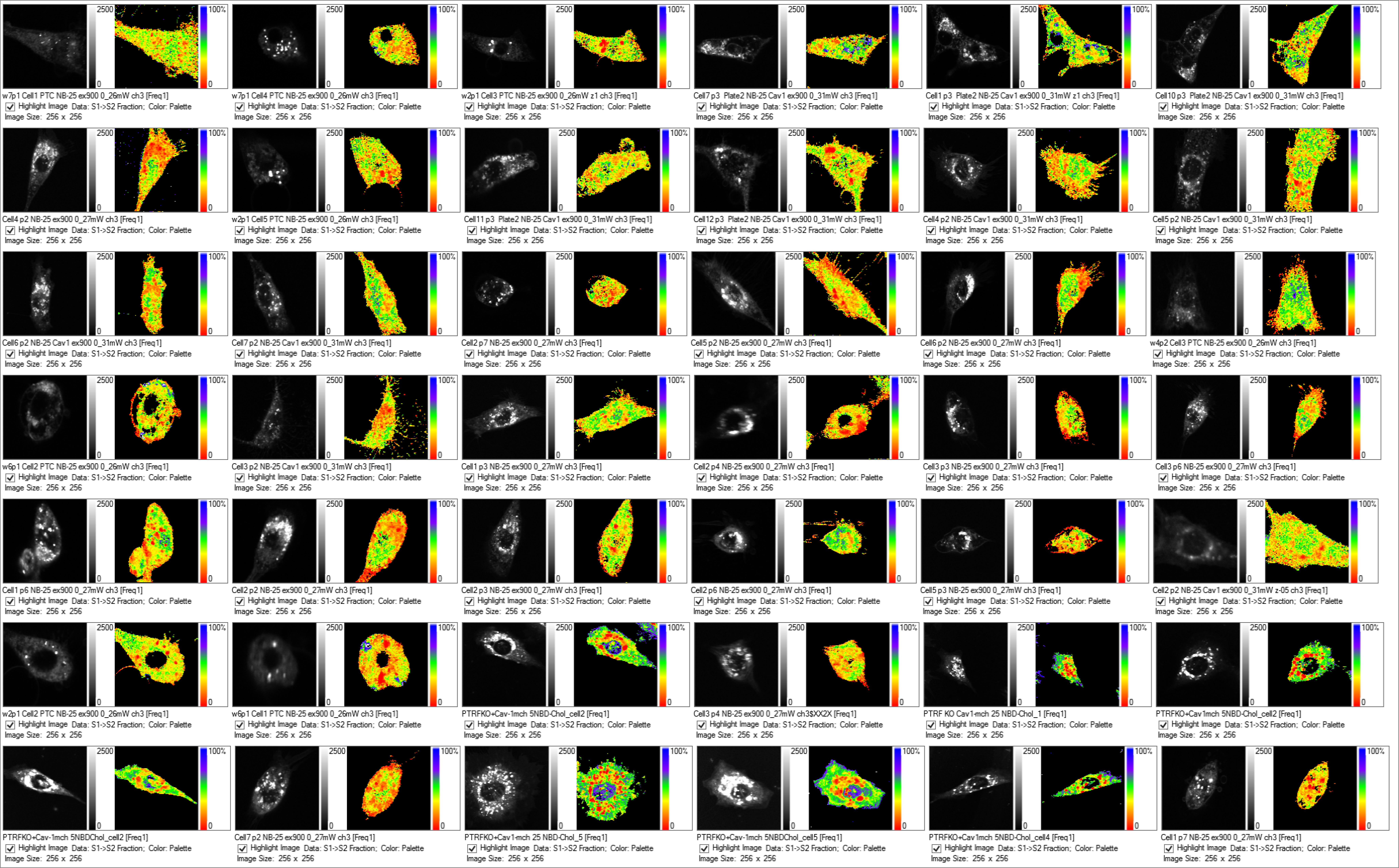

2500
0
100%
0
w7p1 Cell1 PTC NB-25 ex900 0_26mW ch3 [Freq1]
Highlight Image Data: S1->S2 Fraction; Color: Palette
Image Size: 256 x 256
2500
0
100%
0
w7p1 Cell4 PTC NB-25 ex900 0_26mW ch3 [Freq1]
Highlight Image Data: S1->S2 Fraction; Color: Palette
Image Size: 256 x 256
2500
0
100%
0
w2p1 Cell3 PTC NB-25 ex900 0_26mW z1 ch3 [Freq1]
Highlight Image Data: S1->S2 Fraction; Color: Palette
Image Size: 256 x 256
2500
0
100%
0
Cell7 p3 Plate2 NB-25 Cav1 ex900 0_31mW ch3 [Freq1]
Highlight Image Data: S1->S2 Fraction; Color: Palette
Image Size: 256 x 256
2500
0
100%
0
Cell1 p3 Plate2 NB-25 Cav1 ex900 0_31mW z1 ch3 [Freq1]
Highlight Image Data: S1->S2 Fraction; Color: Palette
Image Size: 256 x 256
2500
0
100%
0
Cell10 p3 Plate2 NB-25 Cav1 ex900 0_31mW ch3 [Freq1]
Highlight Image Data: S1->S2 Fraction; Color: Palette
Image Size: 256 x 256
2500
0
100%
0
Cell4 p2 NB-25 ex900 0_27mW ch3 [Freq1]
Highlight Image Data: S1->S2 Fraction; Color: Palette
Image Size: 256 x 256
2500
0
100%
0
w2p1 Cell5 PTC NB-25 ex900 0_26mW ch3 [Freq1]
Highlight Image Data: S1->S2 Fraction; Color: Palette
Image Size: 256 x 256
2500
0
100%
0
Cell11 p3 Plate2 NB-25 Cav1 ex900 0_31mW ch3 [Freq1]
Highlight Image Data: S1->S2 Fraction; Color: Palette
Image Size: 256 x 256
2500
0
100%
0
Cell12 p3 Plate2 NB-25 Cav1 ex900 0_31mW ch3 [Freq1]
Highlight Image Data: S1->S2 Fraction; Color: Palette
Image Size: 256 x 256
2500
0
100%
0
Cell4 p2 NB-25 Cav1 ex900 0_31mW ch3 [Freq1]
Highlight Image Data: S1->S2 Fraction; Color: Palette
Image Size: 256 x 256
2500
0
100%
0
Cell5 p2 NB-25 Cav1 ex900 0_31mW ch3 [Freq1]
Highlight Image Data: S1->S2 Fraction; Color: Palette
Image Size: 256 x 256
2500
0
100%
0
Cell6 p2 NB-25 Cav1 ex900 0_31mW ch3 [Freq1]
Highlight Image Data: S1->S2 Fraction; Color: Palette
Image Size: 256 x 256
2500
0
100%
0
Cell7 p2 NB-25 Cav1 ex900 0_31mW ch3 [Freq1]
Highlight Image Data: S1->S2 Fraction; Color: Palette
Image Size: 256 x 256
2500
0
100%
0
Cell2 p7 NB-25 ex900 0_27mW ch3 [Freq1]
Highlight Image Data: S1->S2 Fraction; Color: Palette
Image Size: 256 x 256
2500
0
100%
0
Cell5 p2 NB-25 ex900 0_27mW ch3 [Freq1]
Highlight Image Data: S1->S2 Fraction; Color: Palette
Image Size: 256 x 256
2500
0
100%
0
Cell6 p2 NB-25 ex900 0_27mW ch3 [Freq1]
Highlight Image Data: S1->S2 Fraction; Color: Palette
Image Size: 256 x 256
2500
0
100%
0
w4p2 Cell3 PTC NB-25 ex900 0_26mW ch3 [Freq1]
Highlight Image Data: S1->S2 Fraction; Color: Palette
Image Size: 256 x 256
2500
0
100%
0
w6p1 Cell2 PTC NB-25 ex900 0_26mW ch3 [Freq1]
Highlight Image Data: S1->S2 Fraction; Color: Palette
Image Size: 256 x 256
2500
0
100%
0
Cell3 p2 NB-25 Cav1 ex900 0_31mW ch3 [Freq1]
Highlight Image Data: S1->S2 Fraction; Color: Palette
Image Size: 256 x 256
2500
0
100%
0
Cell1 p3 NB-25 ex900 0_27mW ch3 [Freq1]
Highlight Image Data: S1->S2 Fraction; Color: Palette
Image Size: 256 x 256
2500
0
100%
0
Cell2 p4 NB-25 ex900 0_27mW ch3 [Freq1]
Highlight Image Data: S1->S2 Fraction; Color: Palette
Image Size: 256 x 256
2500
0
100%
0
Cell3 p3 NB-25 ex900 0_27mW ch3 [Freq1]
Highlight Image Data: S1->S2 Fraction; Color: Palette
Image Size: 256 x 256
2500
0
100%
0
Cell3 p6 NB-25 ex900 0_27mW ch3 [Freq1]
Highlight Image Data: S1->S2 Fraction; Color: Palette
Image Size: 256 x 256
2500
0
100%
0
Cell1 p6 NB-25 ex900 0_27mW ch3 [Freq1]
Highlight Image Data: S1->S2 Fraction; Color: Palette
Image Size: 256 x 256
2500
0
100%
0
Cell2 p2 NB-25 ex900 0_27mW ch3 [Freq1]
Highlight Image Data: S1->S2 Fraction; Color: Palette
Image Size: 256 x 256
2500
0
100%
0
Cell2 p3 NB-25 ex900 0_27mW ch3 [Freq1]
Highlight Image Data: S1->S2 Fraction; Color: Palette
Image Size: 256 x 256
2500
0
100%
0
Cell2 p6 NB-25 ex900 0_27mW ch3 [Freq1]
Highlight Image Data: S1->S2 Fraction; Color: Palette
Image Size: 256 x 256
2500
0
100%
0
Cell5 p3 NB-25 ex900 0_27mW ch3 [Freq1]
Highlight Image Data: S1->S2 Fraction; Color: Palette
Image Size: 256 x 256
2500
0
100%
0
Cell2 p2 NB-25 Cav1 ex900 0_31mW z-05 ch3 [Freq1]
Highlight Image Data: S1->S2 Fraction; Color: Palette
Image Size: 256 x 256
2500
0
100%
0
w2p1 Cell2 PTC NB-25 ex900 0_26mW ch3 [Freq1]
Highlight Image Data: S1->S2 Fraction; Color: Palette
Image Size: 256 x 256
2500
0
100%
0
w6p1 Cell1 PTC NB-25 ex900 0_26mW ch3 [Freq1]
Highlight Image Data: S1->S2 Fraction; Color: Palette
Image Size: 256 x 256
2500
0
100%
0
PTRFKO+Cav-1mch 5NBD-Chol_cell2 [Freq1]
Highlight Image Data: S1->S2 Fraction; Color: Palette
Image Size: 256 x 256
2500
0
100%
0
Cell3 p4 NB-25 ex900 0_27mW ch3$XX2X [Freq1]
Highlight Image Data: S1->S2 Fraction; Color: Palette
Image Size: 256 x 256
2500
0
100%
0
PTRF KO Cav1-mch 25 NBD-Chol_1 [Freq1]
Highlight Image Data: S1->S2 Fraction; Color: Palette
Image Size: 256 x 256
2500
0
100%
0
PTRFKO+Cav1mch 5NBD-Chol_cell2 [Freq1]
Highlight Image Data: S1->S2 Fraction; Color: Palette
Image Size: 256 x 256
2500
0
100%
0
PTRFKO+Cav-1mch 5NBDChol_cell2 [Freq1]
Highlight Image Data: S1->S2 Fraction; Color: Palette
Image Size: 256 x 256
2500
0
100%
0
Cell7 p2 NB-25 ex900 0_27mW ch3 [Freq1]
Highlight Image Data: S1->S2 Fraction; Color: Palette
Image Size: 256 x 256
2500
0
100%
0
PTRFKO+Cav1-mch 25 NBD-Chol_5 [Freq1]
Highlight Image Data: S1->S2 Fraction; Color: Palette
Image Size: 256 x 256
2500
0
100%
0
PTRFKO+Cav-1mch 5NBDChol_cell5 [Freq1]
Highlight Image Data: S1->S2 Fraction; Color: Palette
Image Size: 256 x 256
2500
0
100%
0
PTRFKO+Cav1mch 5NBD-Chol_cell4 [Freq1]
Highlight Image Data: S1->S2 Fraction; Color: Palette
Image Size: 256 x 256
2500
0
100%
0
Cell1 p7 NB-25 ex900 0_27mW ch3 [Freq1]
Highlight Image Data: S1->S2 Fraction; Color: Palette
Image Size: 256 x 256

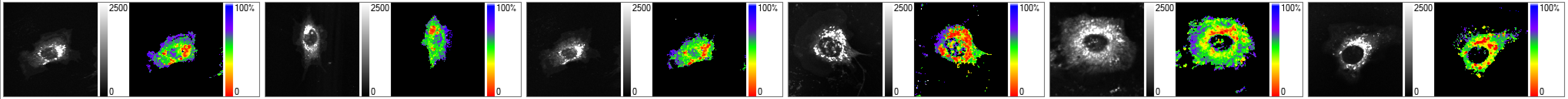

PTRF KO PTRF-mch 25 NBD-Chol_2 [Freq1]
☑ Highlight Image Data: S1->S2 Fraction; Color: Palette
Image Size: 256 x 256

PTRF KO PTRF-mch 25 NBD-Chol_3 [Freq1]
☑ Highlight Image Data: S1->S2 Fraction; Color: Palette
Image Size: 256 x 256

PTRF KO PTRF-mch 25 NBD-Chol_5 [Freq1]
☑ Highlight Image Data: S1->S2 Fraction; Color: Palette
Image Size: 256 x 256

PTRFko+PTRF-mch 25 NBD-Chol_4 [Freq1]
☑ Highlight Image Data: S1->S2 Fraction; Color: Palette
Image Size: 256 x 256

PTRFKO+PTRF 25NBD-Chol_cell1 [Freq1]
☑ Highlight Image Data: S1->S2 Fraction; Color: Palette
Image Size: 256 x 256

PTRFKO+PTRFmcher_cell1 [Freq1]
☑ Highlight Image Data: S1->S2 Fraction; Color: Palette
Image Size: 256 x 256

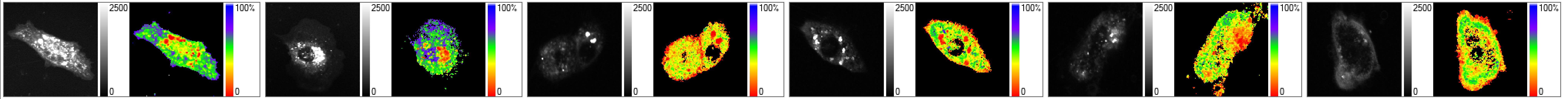

PTRFKO+PTRF-mcher5NBDChol_cell1 [Freq1]
☑ Highlight Image Data: S1->S2 Fraction; Color: Palette
Image Size: 256 x 256

PTRFKO+PTRF-mcher5NBDChol_cell5 [Freq1]
☑ Highlight Image Data: S1->S2 Fraction; Color: Palette
Image Size: 256 x 256

Cell1 p6 PTRF NB-25 ex900 0_2mW ch3 [Freq1]
☑ Highlight Image Data: S1->S2 Fraction; Color: Palette
Image Size: 256 x 256

Cell4 p6 PTRF NB-25 ex900 0_2mW ch3 [Freq1]
☑ Highlight Image Data: S1->S2 Fraction; Color: Palette
Image Size: 256 x 256

Cell1 p7 PTRF NB-25 ex900 0_2mW z-3 ch3 [Freq1]
☑ Highlight Image Data: S1->S2 Fraction; Color: Palette
Image Size: 256 x 256

Cell2 p7 PTRF NB-25 ex900 0_2mW ch3 [Freq1]
☑ Highlight Image Data: S1->S2 Fraction; Color: Palette
Image Size: 256 x 256

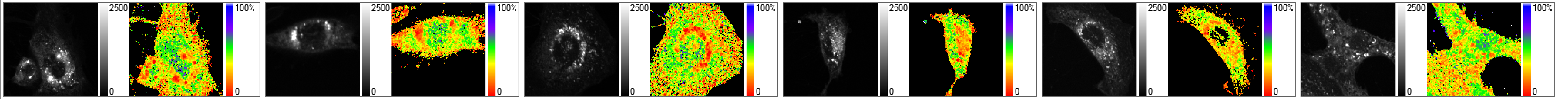

Cell2 p8 PTRF NB-25 ex900 0_2mW ch3 [Freq1]
☑ Highlight Image Data: S1->S2 Fraction; Color: Palette
Image Size: 256 x 256

Cell4 p8 PTRF NB-25 ex900 0_2mW ch3 [Freq1]
☑ Highlight Image Data: S1->S2 Fraction; Color: Palette
Image Size: 256 x 256

Cell1 w2p1 PTRF NB-25 ex900 0_31mW ch3 [Freq1]
☑ Highlight Image Data: S1->S2 Fraction; Color: Palette
Image Size: 256 x 256

Cell2 w2p1 PTRF NB-25 ex900 0_31mW ch3 [Freq1]
☑ Highlight Image Data: S1->S2 Fraction; Color: Palette
Image Size: 256 x 256

Cell3 w2p1 PTRF NB-25 ex900 0_31mW ch3 [Freq1]
☑ Highlight Image Data: S1->S2 Fraction; Color: Palette
Image Size: 256 x 256

Cell5 w2p1 PTRF NB-25 ex900 0_31mW ch3 [Freq1]
☑ Highlight Image Data: S1->S2 Fraction; Color: Palette
Image Size: 256 x 256

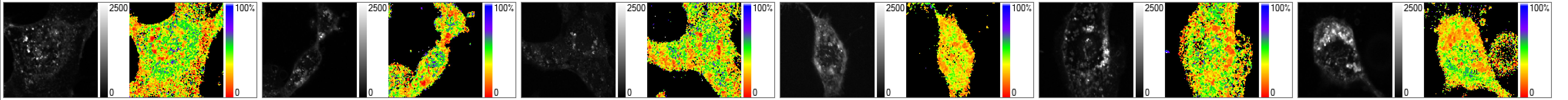

Cell6 w2p1 PTRF NB-25 ex900 0_31mW ch3 [Freq1]
☑ Highlight Image Data: S1->S2 Fraction; Color: Palette
Image Size: 256 x 256

Cell7 w2p1 PTRF NB-25 ex900 0_31mW ch3 [Freq1]
☑ Highlight Image Data: S1->S2 Fraction; Color: Palette
Image Size: 256 x 256

Cell8 w2p1 PTRF NB-25 ex900 0_31mW z-05 ch3 [Freq1]
☑ Highlight Image Data: S1->S2 Fraction; Color: Palette
Image Size: 256 x 256

Cell9 w2p1 PTRF NB-25 ex900 0_31mW ch3 [Freq1]
☑ Highlight Image Data: S1->S2 Fraction; Color: Palette
Image Size: 256 x 256

Cell10 w2p1 PTRF NB-25 ex900 0_31mW ch3 [Freq1]
☑ Highlight Image Data: S1->S2 Fraction; Color: Palette
Image Size: 256 x 256

Cell1 w7p1 PTRF NB-25 ex900 0_31mW ch3$XX2X [Freq1]
☑ Highlight Image Data: S1->S2 Fraction; Color: Palette
Image Size: 256 x 256

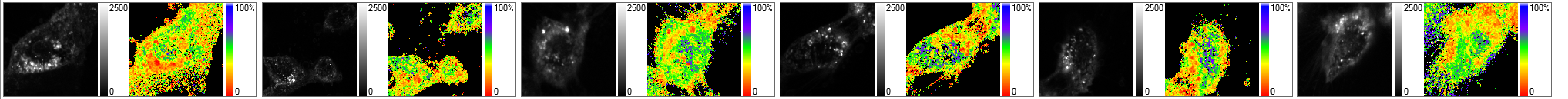

Cell2 w7p1 PTRF NB-25 ex900 0_31mW ch3 [Freq1]
☑ Highlight Image Data: S1->S2 Fraction; Color: Palette
Image Size: 256 x 256

Cell3 w7p1 PTRF NB-25 ex900 0_31mW ch3 [Freq1]
☑ Highlight Image Data: S1->S2 Fraction; Color: Palette
Image Size: 256 x 256

Cell2 w8p1 PTRF NB-25 ex900 0_31mW ch3 [Freq1]
☑ Highlight Image Data: S1->S2 Fraction; Color: Palette
Image Size: 256 x 256

Cell4 w8p1 PTRF NB-25 ex900 0_31mW ch3 [Freq1]
☑ Highlight Image Data: S1->S2 Fraction; Color: Palette
Image Size: 256 x 256

Cell5 w8p1 PTRF NB-25 ex900 0_31mW ch3 [Freq1]
☑ Highlight Image Data: S1->S2 Fraction; Color: Palette
Image Size: 256 x 256

Cell6 w8p1 PTRF NB-25 ex900 0_31mW ch3 [Freq1]
☑ Highlight Image Data: S1->S2 Fraction; Color: Palette
Image Size: 256 x 256

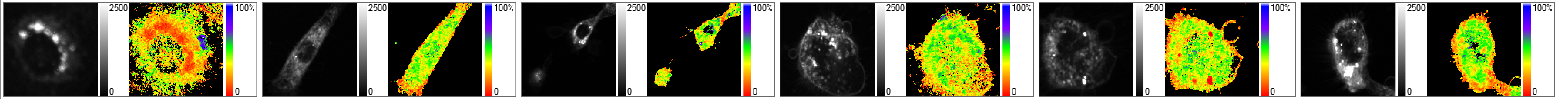

Cell4 w2p2 PTRF NB-25 ex900 0_31mW z-1 ch3 [Freq1]
☑ Highlight Image Data: S1->S2 Fraction; Color: Palette
Image Size: 256 x 256

w2p1 Cell1 PTRF NB-25 ex900 0_25mW ch3 [Freq1]
☑ Highlight Image Data: S1->S2 Fraction; Color: Palette
Image Size: 256 x 256

w2p1 Cell5 PTRF NB-25 ex900 0_25mW z1 ch3 [Freq1]
☑ Highlight Image Data: S1->S2 Fraction; Color: Palette
Image Size: 256 x 256

w7p1 Cell2 PTRF NB-25 ex900 0_27mW ch3 [Freq1]
☑ Highlight Image Data: S1->S2 Fraction; Color: Palette
Image Size: 256 x 256

w8p1 Cell2 PTRF NB-25 ex900 0_25mW ch3 [Freq1]
☑ Highlight Image Data: S1->S2 Fraction; Color: Palette
Image Size: 256 x 256

w8p1 Cell3 PTRF NB-25 ex900 0_25mW ch3 [Freq1]
☑ Highlight Image Data: S1->S2 Fraction; Color: Palette
Image Size: 256 x 256

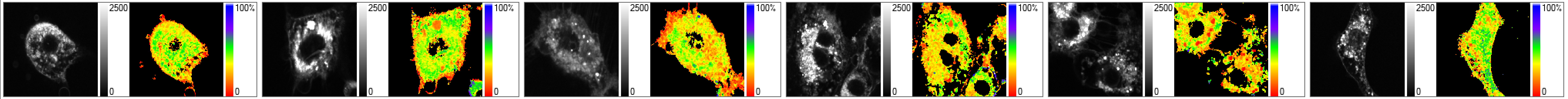

w8p1 Cell5 PTRF NB-25 ex900 0_27mW ch3 [Freq1]
☑ Highlight Image Data: S1->S2 Fraction; Color: Palette
Image Size: 256 x 256

w3p2 Cell5 PTRF NB-25 ex900 0_25mW ch3 [Freq1]
☑ Highlight Image Data: S1->S2 Fraction; Color: Palette
Image Size: 256 x 256

w3p2 Cell7 PTRF NB-25 ex900 0_25mW ch3 [Freq1]
☑ Highlight Image Data: S1->S2 Fraction; Color: Palette
Image Size: 256 x 256

w3p2 Cell8 PTRF NB-25 ex900 0_25mW ch3 [Freq1]
☑ Highlight Image Data: S1->S2 Fraction; Color: Palette
Image Size: 256 x 256

w3p2 Cell9 PTRF NB-25 ex900 0_25mW ch3 [Freq1]
☑ Highlight Image Data: S1->S2 Fraction; Color: Palette
Image Size: 256 x 256

w6p2 Cell1 PTRF NB-25 ex900 0_27mW z-1 ch3 [Freq1]
☑ Highlight Image Data: S1->S2 Fraction; Color: Palette
Image Size: 256 x 256

Supplementary Information

Figure legends

Supplementary Figure 1. FLIM PTRFKO.

Gallery containing all the FLIM images from PTRFKO MEFs that were used for quantification in Extended Data Figure 4D.

Supplementary Figure 1. FLIM PTRFKO+Cav1.

Gallery containing all the FLIM images from PTRFKO+Cav1 MEFs that were used for quantification in Extended Data Figure 4D.

Supplementary Figure 1. FLIM PTRFKO+PTRF.

Gallery containing all the FLIM images from PTRFKO+PTRF MEFs that were used for quantification in Extended Data Figure 4D.

Theory supplement

Fidel-Nicolás Lolo et. al

June 11, 2021

1 Effect of Cholesterol decondensation

Caveolin clustering leads to cholesterol enrichment in caveolae (Parton and Simons, 2007). In this section, we examine the hypothesis that cholesterol de-condensation in enriched domains may enable membrane buffering in response to increased tension, Fig. 1(A). Experiments on artificial giant unilamellar vesicles have reported contradictory results in this regard. While mixing of previously phase-separated domains was induced by increasing tension through micropipette aspiration (Portet et al., 2012), suggesting that de-condensation of cholesterol-rich domains can release membrane area, de-mixing and domain formation was induced by osmotic shocks leading to vesicle swelling (Hamada et al., 2011; Oglęcka et al., 2014). To theoretically estimate the degree of membrane area buffering by cholesterol de-condensation, we refer to quantitative studies of the effect of cholesterol concentration on the area per lipid for binary mixtures (Hung et al., 2007; de Meyer and Smit, 2009), although generally more complex lipid mixtures are required for phase separation.

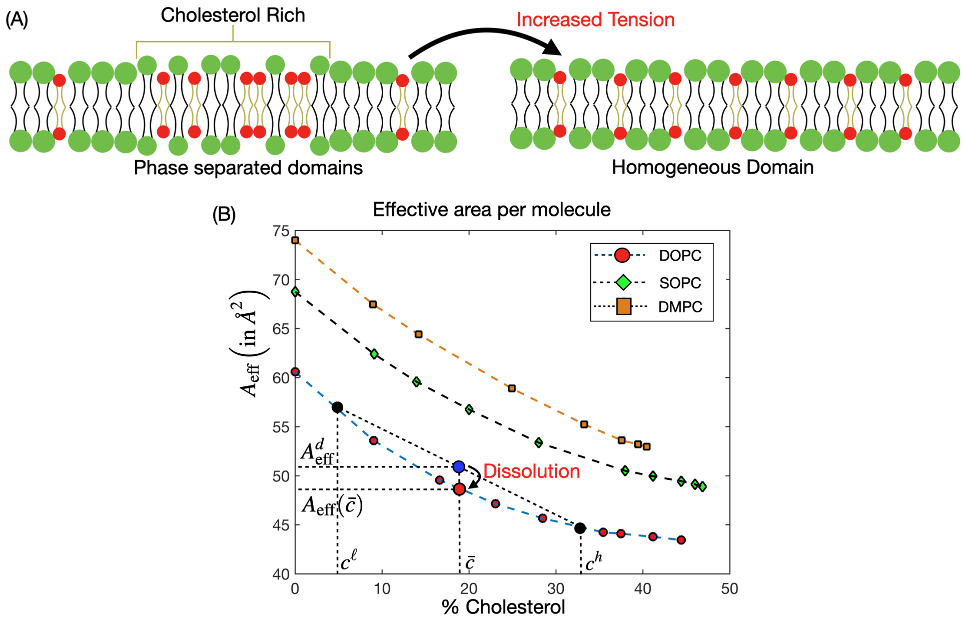

Figure 1: (A) Schematic depiction of the response of a lipid membrane containing cholesterol-rich domains to increased tension. Cholesterol molecules are depicted in red and the other lipids in green. As the cholesterol rich domains disperse, a homogeneous state is attained, (B) average area per lipid molecule (weighted average of non-cholesterol lipids and cholesterol) as a function of molar fraction of cholesterol obtained from Hung et al. (2007). Two mixtures with high and low cholesterol concentrations, c^h and c^ℓ, are represented with black dots. If these two mixtures were different phases co-existing on a membrane, the average area-per-lipid of the phase-separated mixture would be given by the straight line connecting the black dots, for various proportions of each of the phases, and hence for each average cholesterol concentration of $\bar{c}$ (blue dot). If instead, at cholesterol concentration $\bar{c}$ the membrane was fully mixed, the average area per lipid would be given by the red dot. Since the relation between average area per lipid versus cholesterol concentration is convex, the mixed state occupies less area, and hence mixing does not release area.

Hung et al. (2007) reported how increasing cholesterol concentration reduces the average area per lipid molecule for membranes made of of binary mixtures of cholesterol and DOPC, SOPC or DMPC, Fig. 1(B). Assuming a given patch of phase separated membrane having area $\bar{A}$ composed of cholesterol-enriched domains of area A_h at a molar cholesterol concentration c^h and average area per molecule $A_{\text{eff}}(c^h)$ in a cholesterol-depraved matrix at cholesterol concentration c^ℓ and average area per molecule $A_{\text{eff}}(c^\ell)$ as shown in Fig. 1(A) and with the

black dots for DOPC in Fig 1(B). We assume that membrane stretching by an osmotic shock is fast enough so that the number of lipids in the membrane stays constant after stretch application. We obtain the total number of lipids as a summation of lipids in the cholesterol enriched domain and the deprived domain through

$$N_{\text{tot}} = \frac{A_h}{A_{\text{eff}}(c^h)} + \frac{\bar{A} - A_h}{A_{\text{eff}}(c^\ell)}. \tag{1}$$

Out of these, the total number of cholesterol molecules is given by

$$N^c_{\text{tot}} = c^h \frac{A_h}{A_{\text{eff}}(c^h)} + c^\ell \frac{\bar{A} - A_h}{A_{\text{eff}}(c^\ell)}. \tag{2}$$

Hence, the average cholesterol concentration in the system is given by

$$\bar{c} = \frac{N^c_{\text{tot}}}{N_{\text{tot}}} = \alpha c^h + (1 - \alpha)\, c^\ell \tag{3}$$

where

$$\alpha = \frac{A_h A_{\text{eff}}(c^\ell)}{A_h A_{\text{eff}}(c^\ell) + (\bar{A} - A_h) A_{\text{eff}}(c^h)}. \tag{4}$$

is bounded such that $0 < \alpha < 1$. Thus, we can obtain that cholesterol concentration for the mixed phase is bounded above and below by the cholesterol concentrations in enriched and deprived domains such that $c^\ell < \bar{c} < c^h$. The average area per lipid for this concentration $A_{\text{eff}}(\bar{c})$ can be obtained from the data for average area as a function of cholesterol concentration as shown in Fig. 1(B). Now, we have to compare the area per lipid in a mixed system at cholesterol concentration $\bar{c}$, i.e. $A_{\text{eff}}(\bar{c})$, with the average area per lipid in the demixed system A^d_{eff} which can be evaluated by dividing the total area of membrane $\bar{A}$ by the total number of molecules

$$A^d_{\text{eff}} = \frac{\bar{A}}{N_{\text{tot}}} = \alpha A_{\text{eff}}(c^h) + (1 - \alpha) A_{\text{eff}}(c^\ell). \tag{5}$$

This average area lies along the straight line joining average area per molecules for the phase segregated domains at an average cholesterol concentration $\bar{c}$, as shown with blue dot in Fig. 1(B). Comparison of average area per molecule in the homogenous and the phase segregated domains using Fig. 1(B) shows that $A_{\text{eff}}(\bar{c}) < A^d_{\text{eff}}$, i.e. that the average area per molecule should be higher in the de-mixed phase. This inequality holds because the curves in Fig. 1(B) are convex. Thus, rather than providing excess area, cholesterol de-condensation should lead according to this estimation to a reduction in area and hence cannot be the mechanism through which membranes release area in response to stretch. On the contrary we can expect a buffer of area by further de-mixing of phase separated domains. We can generalize this result to all cases where the average area per molecule is a convex function of cholesterol concentration and the total number of molecules is fixed. While the lipid mixtures in real membranes are much more complex, this simple analysis does not support the idea or membrane area buffering by demixing.

2 Model of individual domains of curvature-active proteins

To model individual curved protein domains, we consider the dynamical chemo-mechanical model presented in detail in Tozzi et al. (2019) particularized to axisymmetry. In this model, curvature-active proteins are modeled as a concentration field of a diffusing species impinging a concentration-dependent spontaneous curvature (Sorre et al., 2012), and the interaction between protein enrichment and membrane curvature is accounted for self-consistently. We formalize the dynamics of the system using Onsager's variational principle (Doi, 2011; Arroyo et al., 2018), according to which the system evolves to minimize a functional accounting for the rate of change of free energy, dissipation and external power input.

2.1 Free Energy

The free energy of a patch of membrane (Γ) with local area fraction of curvature-active protein (ϕ) has contributions from the bending energy, the mixing entropy and the self-interaction of proteins

$$\mathcal{F} = \mathcal{F}_{\text{bend}} + \mathcal{F}_{\text{ent}} + \mathcal{F}_{\text{si}}. \tag{6}$$

Here, the bending energy contribution is given by the standard Helfrich energy neglecting the contributions from Gaussian curvature

$$\mathcal{F}_{\text{bend}} = \int_\Gamma \frac{\kappa}{2}(H - \bar{C}\phi)^2 \, dS, \tag{7}$$

with H representing twice the sum of principal curvatures and $\bar{C}$ the spontaneous curvature of the protein-lipid composite at saturation (corresponding to $\phi = 1$). The mixing entropy of proteins is

$$\mathcal{F}_{\text{ent}} = \int_\Gamma \frac{k_B T}{a_p} \left[\phi \log \phi + (1-\phi)\log(1-\phi)\right] dS, \tag{8}$$

where k_B is the Boltzmann constant and a_p is area per molecule. Finally, the self-interaction is assumed to be of the form

$$\mathcal{F}_{\text{si}} = \int_\Gamma \frac{\chi}{2a_p}\phi^2 \, dS + \int_\Gamma \frac{\Lambda}{2a_p}|\nabla_S \phi|^2 \, dS, \tag{9}$$

where the first term models attractive ($\chi < 0$) or repulsive ($\chi > 0$) interactions and the second term regularizes the boundary of protein-rich domains and governs the interfacial energy between domains. The symbol ∇_S denotes here the surface gradient.

2.2 Dissipation

We consider lipid membrane to be inextensible Newtonian surface fluid with rate-of-deformation tensor $\boldsymbol{d}$ and coefficient of viscosity η. Proteins diffuse on this surface with velocity $\boldsymbol{w}$ relative to the membrane and drag coefficient ξ/a_p. This allows us to write the total dissipation potential as

$$\mathcal{D} = \mathcal{D}_{\text{mem}} + \mathcal{D}_{\text{prot}} = \int_\Gamma \eta \boldsymbol{d} : \boldsymbol{d} \, dS + \int_\Gamma \frac{\xi}{2a_p}\phi |\boldsymbol{w}|^2 \, dS. \tag{10}$$

2.3 Power input

In our model, mechanical or chemical power can be provided at the boundary of the membrane patch Γ by fixing membrane tension or the chemical potential of proteins. Here, we consider that tension σ is applied but that the number of proteins in our patch remains fixed with average density is $\bar{\phi}$ and hence the flux of proteins $\boldsymbol{w}$ is zero at the boundary. In this case, the power input is purely mechanical and takes the form of a line integral long the boundary of the patch, $\partial\Gamma$,

$$\mathcal{P} = -\int_{\partial\Gamma} \sigma \boldsymbol{v} \cdot \boldsymbol{\nu} \, d\ell, \tag{11}$$

where $\boldsymbol{v}$ is the membrane velocity and $\boldsymbol{\nu}$ is the outer normal to $\partial\Gamma$ tangential to the surface.

2.4 Governing equations and numerical solution

We form the Rayleighian functional as $\mathcal{R} = \dot{\mathcal{F}} + \mathcal{D} + \mathcal{P}$ an minimize it with respect to $\boldsymbol{v}$ and $\boldsymbol{w}$ subject to mass conservation of membrane proteins and membrane inextensibility to obtain the transport and mechanical governing equations. These equations are solved under the hypothesis of axisymmetry using B-Spline approximations as described in detail in Tozzi et al. (2019).

2.5 Spontaneous phase separation

A flat patch of membrane with uniform distribution of proteins $\bar{\phi}$ and applied tension $\sigma > 0$ can become unstable, and hence lead to the formation of a protein-rich domain, by two different mechanisms. First, domains can form if the net enthalpic and entropic protein interactions become attractive (Tozzi et al., 2019)

$$a^{\text{eff}} = \chi + a_p \kappa \bar{C}^2 + \frac{k_B T}{\bar{\phi}(1-\bar{\phi})} < 0, \tag{12}$$

resulting in a purely chemical phase separation. Second, a tension-dependent chemo-mechanical instability can occur if

$$\kappa \bar{C} - \sqrt{\kappa (a^{\text{eff}}/a_p)} \geq \sqrt{\sigma^{\text{eff}} \Lambda / a_p}, \tag{13}$$

where $\sigma^{\text{eff}} = \sigma + \frac{k_B T}{a_p}\log(1-\bar{\phi}) - \frac{\chi}{2a_p}\bar{\phi}^2 - \frac{\kappa}{2}\bar{C}^2\bar{\phi}^2$. In both cases, the resulting protein-rich domains are curved if $\bar{C}$ is different from zero, and hence they will both respond to a sufficiently large increase in tension by flattening and releasing area. However, while in the first mechanism protein-rich domains will remain upon tension application since the criterion for domain formation is independent of tension, in the second case these domains will dissolve and release previously clustered proteins to the membrane. This situation is summarized in Fig. 2. Since the second case is consistent with the tension-induced disassembly of Cav1 domains observed experimentally with reduced membrane order as shown in Suppl. Figure 5E and F, we choose the model parameters so that Eq. (12) is not satisfied but Eq. (13) is satisfied for low membrane tensions and for both the Cav1 and the PTRF models referred to in the main text. All the model parameters used are given in Table 1 unless otherwise stated.

Table 1: Value of model parameters

Parameter	Value
Bending Stiffness, κ	20 k_BT
Membrane viscosity, η	$5 \cdot 10^{-9}$ Ns/m
Drag coefficient, ξ/a_p	$3 \cdot 10^{8}$ Ns/m^3
Area per protein, a_p	100 nm^2
Spontaneous curvature of Cav1 model, $\bar{C}$	2/200 nm^{-1}
Spontaneous curvature of PTRF model, $\bar{C}$	2/50 nm^{-1}
Self-interaction of proteins, χ/a_p	-0.26 mN/m
Λ/a_p	15 k_BT

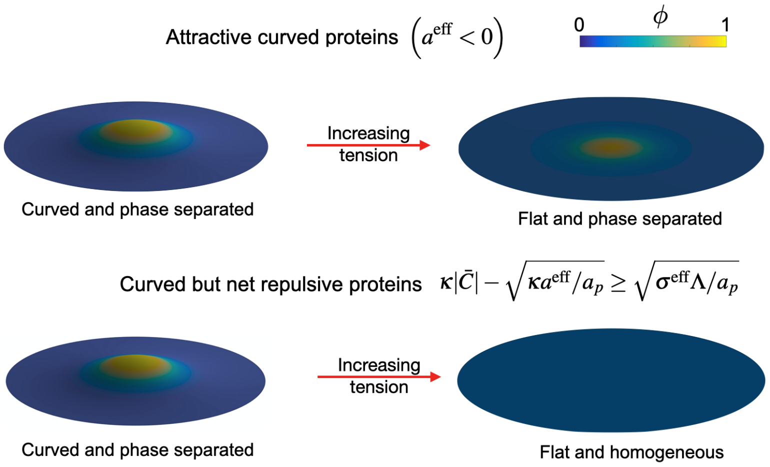

Figure 2: Dissolution of buds in response to stretch depends on the interaction between Cav1 molecules. If net attractive, stretch leads to flattened but phase separated domain whereas, if net repulsive but phase-separated due to the chemo-mechanical mechanism of Eq. (13), stretch leads to a flat and homogeneous state of the membrane. To achieve $a^{\text{eff}} < 0$, in the top panel we have used $\chi/a_p = -0.4$ mN/m.

3 Thermodynamic model for an ensemble of domains

The previous model is able to describe the dynamics of formation and flattening of a single curved protein-rich domain with great detail. However, its 3D implementation to study the collective behavior of an ensemble of domains is extremely challenging. Instead, we develop here a coarse-grained thermodynamical model in which the geometry of domains is simplified to spherical caps but which allows for a varying number density of such domains. In this model, given the average area fraction of proteins ($\bar{\phi}$) and the surface tension (σ), we solve for the number density of domains ρ, their protein coverage ϕ_d, radius R and contact angle ψ, and the protein coverage in the flat membrane domain ϕ_f. We do this by minimizing a free energy accounting for the same contributions as in the previous model, except for a different implementation of interfacial energy between domains. In the previous model, the energy of the diffuse interface was controlled by the second term in Eq. (9) whereas here it is written in terms of the line tension γ of a sharp interface (Reinhard Lipowsky, 1992; Sens and Turner, 2006). Geometric arguments shows that the membrane area fraction of budded domains is $\alpha_d = 2\pi\rho R^2(1-\cos\psi)$ and that of the flat domain $\alpha_f = 1-\alpha_d$. Similarly, the radius of the interface between budded and flat domains is $R\sin\psi$. The free energy per unit area of membrane is then

$$\mathcal{F}(\rho,\phi_d,\phi_f,R,\psi) = \frac{\kappa}{2}(2/R-\bar{C}\phi_d)^2\alpha_d + \mu(\phi_d)\alpha_d + 2\pi\gamma\rho R\sin\psi + \frac{\kappa}{2}\bar{C}^2\phi_f^2\alpha_f + \mu(\phi_f)\alpha_f + \mathcal{F}_{\text{ext}}, \tag{14}$$

where the chemical potential of proteins in the bud and flat domains are denoted by $\mu(\phi_d)$ and $\mu(\phi_f)$ and given by the expression

$$\mu(\phi) = \mu_{a,0}\phi + \frac{k_BT}{a_p}[\phi\log\phi + (1-\phi)\log(1-\phi)] + \frac{\chi}{2a_p}\phi^2. \tag{15}$$

$\mathcal{F}_{\text{ext}}$ denotes the work done by surface tension σ against changes in projected area, and is given by

$$\mathcal{F}_{\text{ext}} = -\sigma\left[\pi\rho(R\sin\psi)^2 - 1\right] \tag{16}$$

We assume that total number of proteins with average area fraction $\bar{\phi}$ is fixed, and hence $\alpha_d\phi_d+\alpha_f\phi_f = 1$. Hence, using this constraint one of the protein area fractions, say ϕ_f, can be expressed in terms of other variables and

the mass-conservation constrained free energy depends only on ρ, ϕ_d, R, and ψ, which we numerically minimize to obtain equilibrium states in terms of the number density, geometry and protein enrichment of domain given $\bar{\phi}$ and σ.

To illustrate the comparison between two models, we considered with the thermodynamic model the specific case where ρ is fixed, mimicking the ensemble of the continuum model of the previous section, and examining the equilibrium states predicted by these two different models for the same model parameters and where the line tension parameter, $\gamma = 0.07$ pN for the Cav1 model and 1.24 pN for the PTRF model, was estimated integrating the free energy density of the continuum model along the diffuse interface and dividing it by its perimeter. Figure 3 shows a good agreement, albeit the geometrical simplification of the second model.

The material parameters used in Fig. 6C coincide with those of the the axisymmetric model in Table 1, except for the fact that Λ is not used in the thermodynamic model and the line tension parameter is chosen as mentioned above.

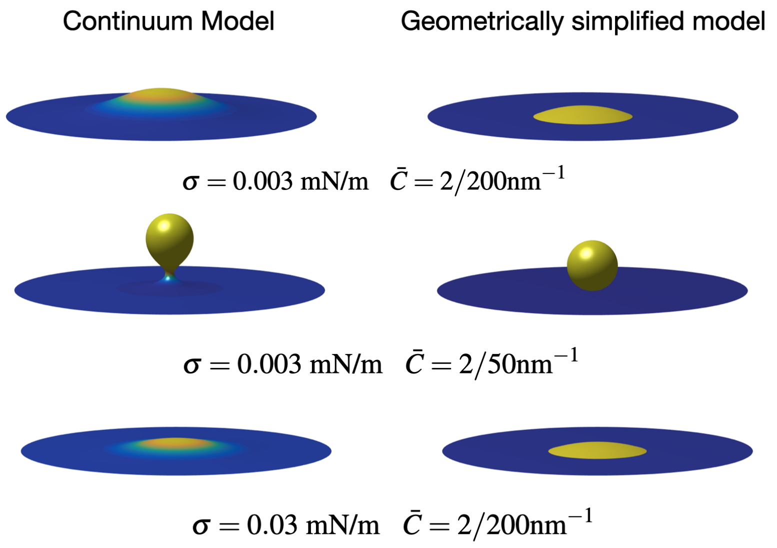

Figure 3: Comparison between the two models for various surface tensions and spontaneous curvatures.

In Fig. 6, we have examined the consequences of the distinct mechanical behavior for ensembles of caveolae and dolines. Our observations show that both structures coexist on the plasma membrane as shown in Fig 5. According to our model, a membrane endowed with both of these area-buffering structures should first respond to an increase of membrane tension taking advantage of the doline system and splitting and flattening these structures, and only in cases of extreme tension or large stretch would cells require Caveolae to release membrane area.

References

Marino Arroyo, Nikhil Walani, Alejandro Torres-Sánchez, and Dimitri Kaurin. Onsager's variational principle in soft matter: Introduction and application to the dynamics of adsorption of proteins onto fluid membranes. In *The Role of Mechanics in the Study of Lipid Bilayers*, pages 287–332. Springer, 2018.

Frédérick de Meyer and Berend Smit. Effect of cholesterol on the structure of a phospholipid bilayer. *Proceedings of the National Academy of Sciences*, 106(10):3654–3658, 2009.

Masao Doi. Onsager's variational principle in soft matter. *Journal of Physics: Condensed Matter*, 23(28):284118, 2011.

Tsutomu Hamada, Yuko Kishimoto, Takeshi Nagasaki, and Masahiro Takagi. Lateral phase separation in tense membranes. *Soft Matter*, 7(19):9061–9068, 2011.

Wei-Chin Hung, Ming-Tao Lee, Fang-Yu Chen, and Huey W Huang. The condensing effect of cholesterol in lipid bilayers. *Biophysical journal*, 92(11):3960–3967, 2007.

Kamila Oglęcka, Padmini Rangamani, Bo Liedberg, Rachel S Kraut, and Atul N Parikh. Oscillatory phase separation in giant lipid vesicles induced by transmembrane osmotic differentials. *Elife*, 3:e03695, 2014.

Robert G Parton and Kai Simons. The multiple faces of caveolae. *Nature reviews Molecular cell biology*, 8(3): 185–194, 2007.

Thomas Portet, Sharona E Gordon, and Sarah L Keller. Increasing membrane tension decreases miscibility temperatures; an experimental demonstration via micropipette aspiration. *Biophysical journal*, 103(8):L35–L37, 2012.

Reinhard Lipowsky. Budding of membranes induced by intramembrane domains. *J. Phys. II France*, 2(10): 1825–1840, 1992.

Pierre Sens and Matthew S Turner. Budded membrane microdomains as tension regulators. *Physical Review E*, 73(3):031918, 2006.

Benoît Sorre, Andrew Callan-Jones, John Manzi, Bruno Goud, Jacques Prost, Patricia Bassereau, and Aurélien Roux. Nature of curvature coupling of amphiphysin with membranes depends on its bound density. *Proceedings of the National Academy of Sciences*, 109(1):173–178, 2012.

Caterina Tozzi, Nikhil Walani, and Marino Arroyo. Out-of-equilibrium mechanochemistry and self-organization of fluid membranes interacting with curved proteins. *New journal of physics*, 21(9):093004, 2019.
