# Supplementary material for: Caveolin-1 dolines form a distinct and rapid caveolae-independent mechanoadaptation system
Source: Nat Cell Biol. 2022 Dec 21;25(1):120–33. doi: 10.1038/s41556-022-01034-3 (PMC9859760; doi:10.1038/s41556-022-01034-3)
Supplement: Supplementary file 2 — Reporting Summary [file 41556_2022_1034_MOESM2_ESM.pdf]

nature portfolio

Corresponding author(s): Miguel Ángel del Pozo and Fidel-Nicolás Lolo Romero

Last updated by author(s): Sep 9, 2022

Reporting Summary

Nature Portfolio wishes to improve the reproducibility of the work that we publish. This form provides structure for consistency and transparency in reporting. For further information on Nature Portfolio policies, see our Editorial Policies and the Editorial Policy Checklist.

Statistics

For all statistical analyses, confirm that the following items are present in the figure legend, table legend, main text, or Methods section.

n/a	Confirmed	
☐	☒	The exact sample size (*n*) for each experimental group/condition, given as a discrete number and unit of measurement
☒	☐	A statement on whether measurements were taken from distinct samples or whether the same sample was measured repeatedly
☐	☒	The statistical test(s) used AND whether they are one- or two-sided<br>*Only common tests should be described solely by name; describe more complex techniques in the Methods section.*
☒	☐	A description of all covariates tested
☐	☒	A description of any assumptions or corrections, such as tests of normality and adjustment for multiple comparisons
☐	☒	A full description of the statistical parameters including central tendency (e.g. means) or other basic estimates (e.g. regression coefficient) AND variation (e.g. standard deviation) or associated estimates of uncertainty (e.g. confidence intervals)
☐	☒	For null hypothesis testing, the test statistic (e.g. *F*, *t*, *r*) with confidence intervals, effect sizes, degrees of freedom and *P* value noted<br>*Give P values as exact values whenever suitable.*
☒	☐	For Bayesian analysis, information on the choice of priors and Markov chain Monte Carlo settings
☒	☐	For hierarchical and complex designs, identification of the appropriate level for tests and full reporting of outcomes
☒	☐	Estimates of effect sizes (e.g. Cohen's *d*, Pearson's *r*), indicating how they were calculated

Our web collection on statistics for biologists contains articles on many of the points above.

Software and code

Policy information about availability of computer code

Data collection	LAS AF V 4.0.0. 11706 software; LAS-AF 2.6.0. build 7266 TIRF; Carl Zeiss ZEN, software 2011 64bits; EM902A, Zeiss; Nikon Corp. Software NIS Elements AR 4.30.02. Build 1053 LO, 64 bits; LABVIEW software 2015; MatLab 8.6; VistaVision_x64_V4.2_Build 364 software.
Data analysis	Super-resolution images were analyzed using LAS AF V 4.0.0. 11706 software, ThunderSTORM v1.3 and ImageJ v1.53q; Electron tomograms were computed and segmented using the software IMOD Package (Kremer JR, Mastronarde DN, McIntosh JR. Computer visualization of three-dimensional image data using IMOD. J Struct Biol. 1996, doi: 10.1006/jsbi.1996.0013).; Automated confocal microscopy images were acquired on a Opera HCSII station and analyzed using the Acapella studio image analysis suite (Perkin Elmer, v2.6); FLIM images were analyzed using SimFCS software, LFD, University of California, Irvine, CA and VistaVision_x64_V4.2_Build 364 software; In-silico data was analyzed by Rosetta software suite v3.8 and v3.9 (www.rosettacommons.org); TIRF videos were obtained using LAS-AF 2.6.0. build 7266 software; Optical Stretching data acquisition and analysis was done with a custom-built LABVIEW software(v 2015); Excel and GraphPad Prism (v 7.0) softwares were used to analyze statistical data and Image J was used for particle tracking in magnetic tweezers experiments, confocal and electron microscopy images analysis. A detailed report of the mathematical model can be found as supplementary Note 1.

For manuscripts utilizing custom algorithms or software that are central to the research but not yet described in published literature, software must be made available to editors and reviewers. We strongly encourage code deposition in a community repository (e.g. GitHub). See the Nature Portfolio guidelines for submitting code & software for further information.

Data

Policy information about availability of data

All manuscripts must include a data availability statement. This statement should provide the following information, where applicable:

- Accession codes, unique identifiers, or web links for publicly available datasets
- A description of any restrictions on data availability
- For clinical datasets or third party data, please ensure that the statement adheres to our policy

Previously published data that were re-analyzed here are available under accession code Uniprot ID: ID: P49817, residues2-178 (mature Cav1 protein). We have deposited the following files in Zenodo: dataset from YAP experiments (DOI: 10.5281/zenodo.7061911), script for YAP analysis (DOI: 10.5281/zenodo.7061924), and STORM images set (DOI: 10.5281/zenodo.7062213). Source data are provided with this study. All other data supporting the findings of this study are available from the corresponding author on reasonable request.

Field-specific reporting

Please select the one below that is the best fit for your research. If you are not sure, read the appropriate sections before making your selection.

☒ Life sciences ☐ Behavioural & social sciences ☐ Ecological, evolutionary & environmental sciences

For a reference copy of the document with all sections, see nature.com/documents/nr-reporting-summary-flat.pdf

Life sciences study design

All studies must disclose on these points even when the disclosure is negative.

Sample size	The sample size was set by the clarity of the phenotype. The lower limit of the sample size was set so it was sufficient to observe clearly the differences (p value below 0.05), while the upper limit was set so it was easily reproducible at a reasonable time for an independent lab. In experiments were a tendency was observed with a small sample size, sample size was increased to determine whether the tendency was statistically significant or not. In some cases the tendency was confirmed and validated statistically while in other cases it was not, in which case the sample size was not further increased.
Data exclusions	No data were excluded from the analyses unless otherwise indicated. For STORM analysis: Clusters touching the ROI borders were excluded (as indicated in the manuscript). For FRIL analysis: no strongly scattering data points were excluded but all quantitative evaluation data points were taken into account and averaged to fully represent biological and technical variabilities.
Replication	All attempts at replication were successful for the experiments present in the paper. The number of replicates is indicated in each figure legend.
Randomization	Experiments were not randomized.
Blinding	Experimenter blinding was not possible as acquisition and analysis were performed by the same investigator. Important information (i.e. GFP label) was also required precluding classic randomization and blinding.

Reporting for specific materials, systems and methods

We require information from authors about some types of materials, experimental systems and methods used in many studies. Here, indicate whether each material, system or method listed is relevant to your study. If you are not sure if a list item applies to your research, read the appropriate section before selecting a response.

Materials & experimental systems

n/a	Involved in the study	
☐	☒	Antibodies
☐	☒	Eukaryotic cell lines
☒	☐	Palaeontology and archaeology
☒	☐	Animals and other organisms
☒	☐	Human research participants
☒	☐	Clinical data
☒	☐	Dual use research of concern

Methods

n/a	Involved in the study	
☒	☐	ChIP-seq
☒	☐	Flow cytometry
☒	☐	MRI-based neuroimaging

Antibodies

Antibodies used	The following primary antibodies were used: The following primary antibodies were used: rabbit monoclonal anti-mouse Caveolin-1, D46G3 Cell signalling XP®#3267; rabbit anti-caveolin 1, sc-894, SantaCruz; mouse monoclonal anti YAP, 63.7, sc-101199, Santa Cruz;

monoclonal anti-Flag, M2, Sigma/F1804; rabbit polyclonal anti-mouse PTRF, ab48824, Abcam; mouse monoclonal anti-alpha tubulin, ab7291, Abcam; mouse anti-GFP, cat# 118114460001, Roche; rat monoclonal anti-mouse total beta 1 integrin, clone MB1.2, MAB1997 Millipore; mouse monoclonal Cav1 SIGMA SAB4200216; Ubiquitin (Enzo, ENZ-ABS840-0100).

Validation

- Anti-caveolin-1, D46G3: this antibody is widely used and validated for western blot and immunofluorescence, applications used in this study, as indicated in the manufacturer´s website. This antibody has been previously validated for WB and immunostaining (DOI: 10.1074/jbc.M005448200).
- Anti-caveolin 1, sc-894: this antibody has been previously validated for EM (DOI: 10.7554/elife.29854)
- Anti mouse monoclonal Cav1 SIGMA SAB4200216: this antibody has been previously validated (DOI: 10.18632/oncotarget.15302)
- Anti Ubiquitin (Enzo, ENZ-ABS840-0100): this antibody has been previously validated (DOI: 10.1073/pnas.2114405119)
- Anti-YAP: this antibody has been extensively used in the YAP field (DOI: 10.1038/nature10137).
- Anti-Flag, M2: this antibody is widely used and validated for immunofluorescence as indicated in the manufacturer´s website; here was used for EM and validation is shown in Supplementary Figure 4D-4E.
- Anti-mouse PTRF, ab48824: this antibody is widely used and validated for western blot and immunofluorescence, applications used in this study, as indicated in the manufacturer´s website. This antibody has been previously validated for WB and immunostaining (DOI: 10.1083/jcb.202006178).
- Anti-alpha tubulin, ab7291: this antibody is widely used and validated for western blot, application used in this study, as indicated in the manufacturer´s website. This antibody has been previously validated for WB (DOI: 10.1016/j.redox.2022.102307).
- Anti-GFP, cat# 118114460001: this antibody is widely used and validated for western blot and immunofluorescence, applications used in this study, as indicated in the manufacturer´s website. This antibody has been previously validated for WB (DOI:10.1038/s41467-022-32364-3).
- Anti- total beta 1 integrin, MAB1997: this antibody is widely used and validated for western blot, application used in this study, as indicated in the manufacturer´s website. This antibody has been previously validated for WB (DOI:10.1091/mbc.E14-07-1203).

Eukaryotic cell lines

Policy information about cell lines

Cell line source(s)

PTRFKO mouse embryonic fibroblasts were a kind gift from Prof. Rob Parton and were originally developed in Paul Pilch's lab doi.org/10.1016/j.cmet.2008.07.008. SH-Sy5y neuroblastoma cell line were a kind gift from Dr. Sergio Casas Tintó (Cajal Institute, Madrid, Spain) and were originally obtained from ATCC.

Authentication

None of the cell lines were autheticated.

Mycoplasma contamination

All cell lines were screened for mycoplasma presence (with Mycoalert PLUS mycoplasma detection kit, Lonza) and the results were negative.

Commonly misidentified lines
(See ICLAC register)

None.
