# Supplementary figures and images for: Caveolin-1 dolines form a distinct and rapid caveolae-independent mechanoadaptation system
Source: Nat Cell Biol. 2022 Dec 21;25(1):120–33. doi: 10.1038/s41556-022-01034-3 (PMC9859760; doi:10.1038/s41556-022-01034-3)

From figure 7

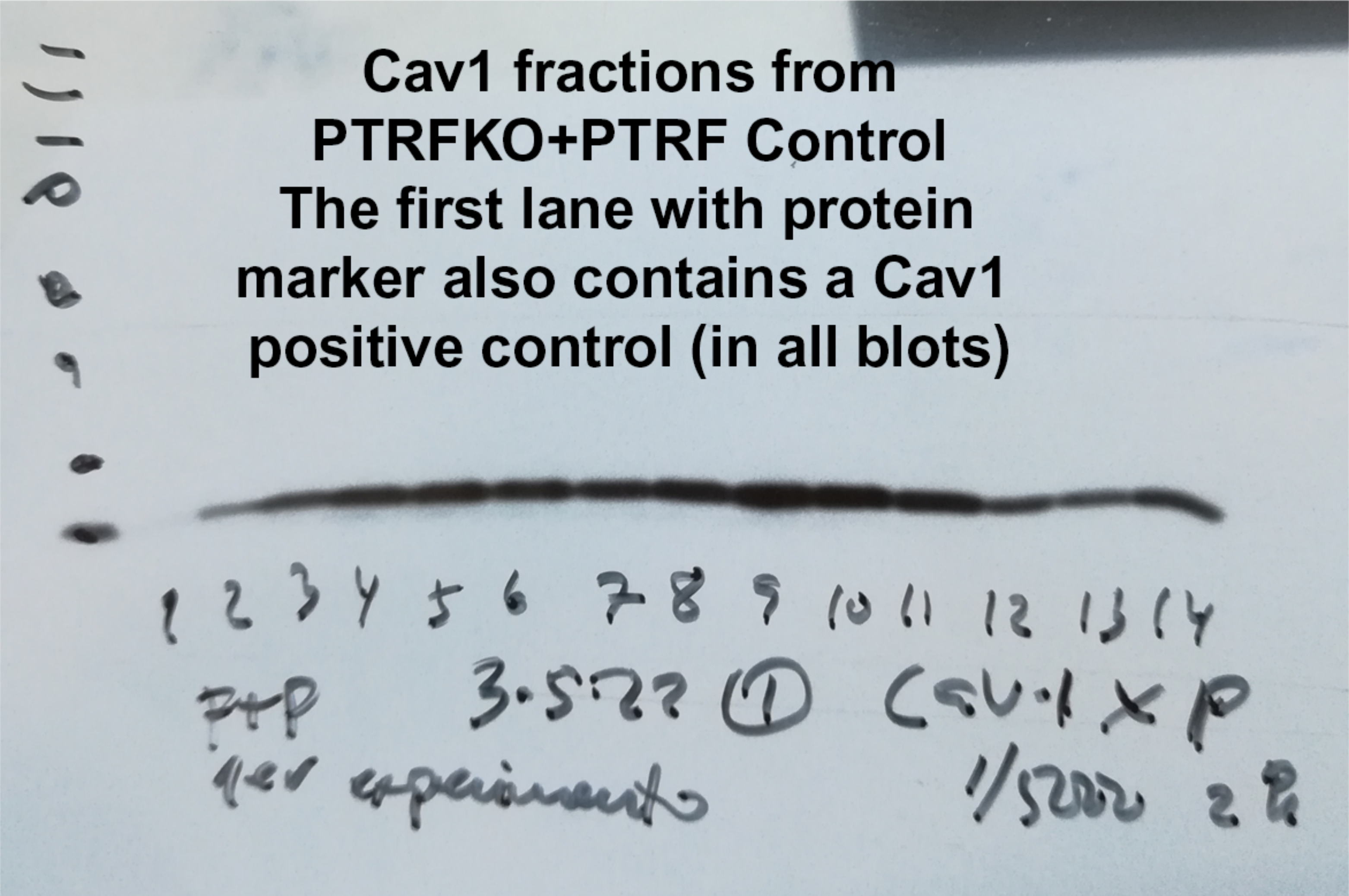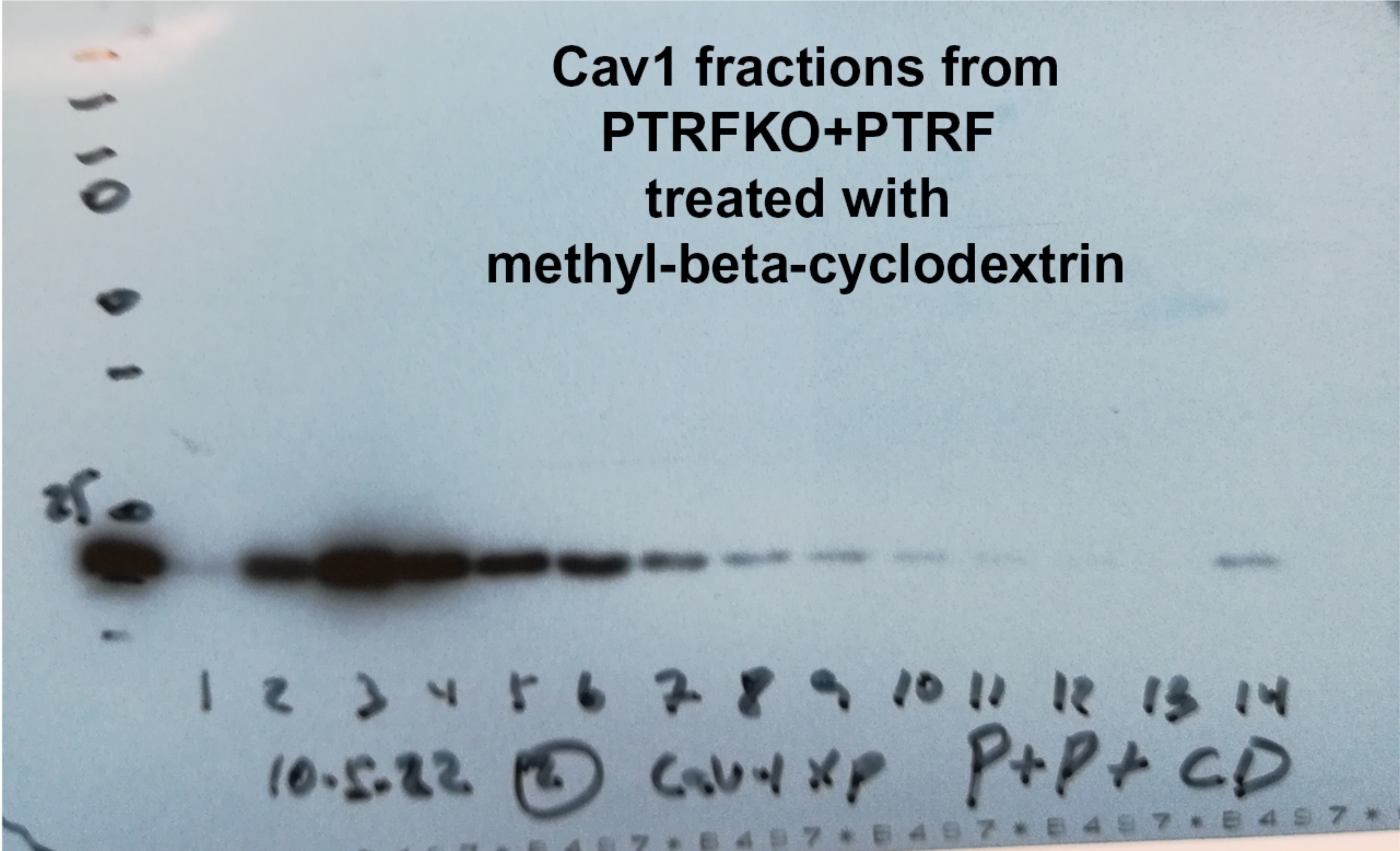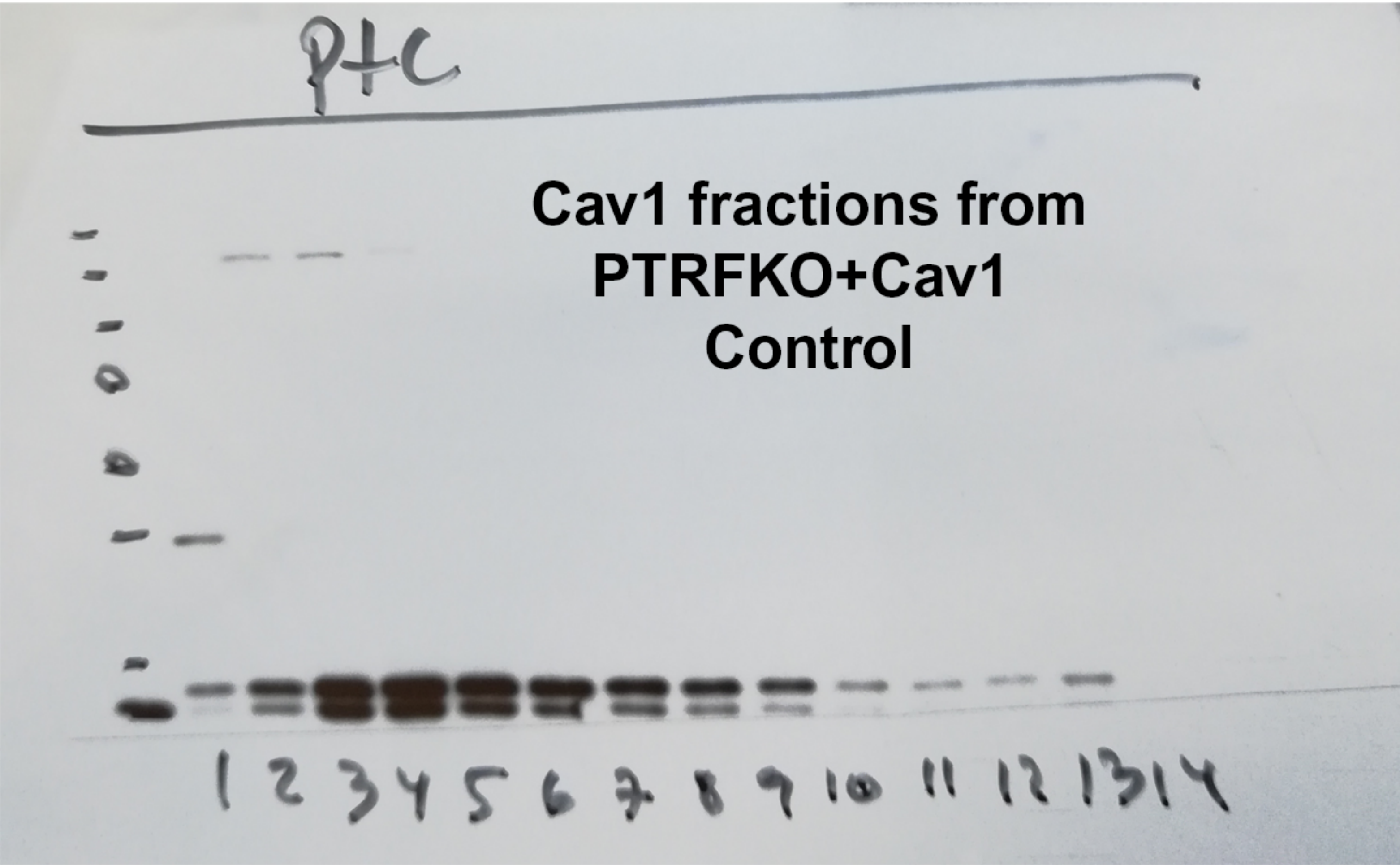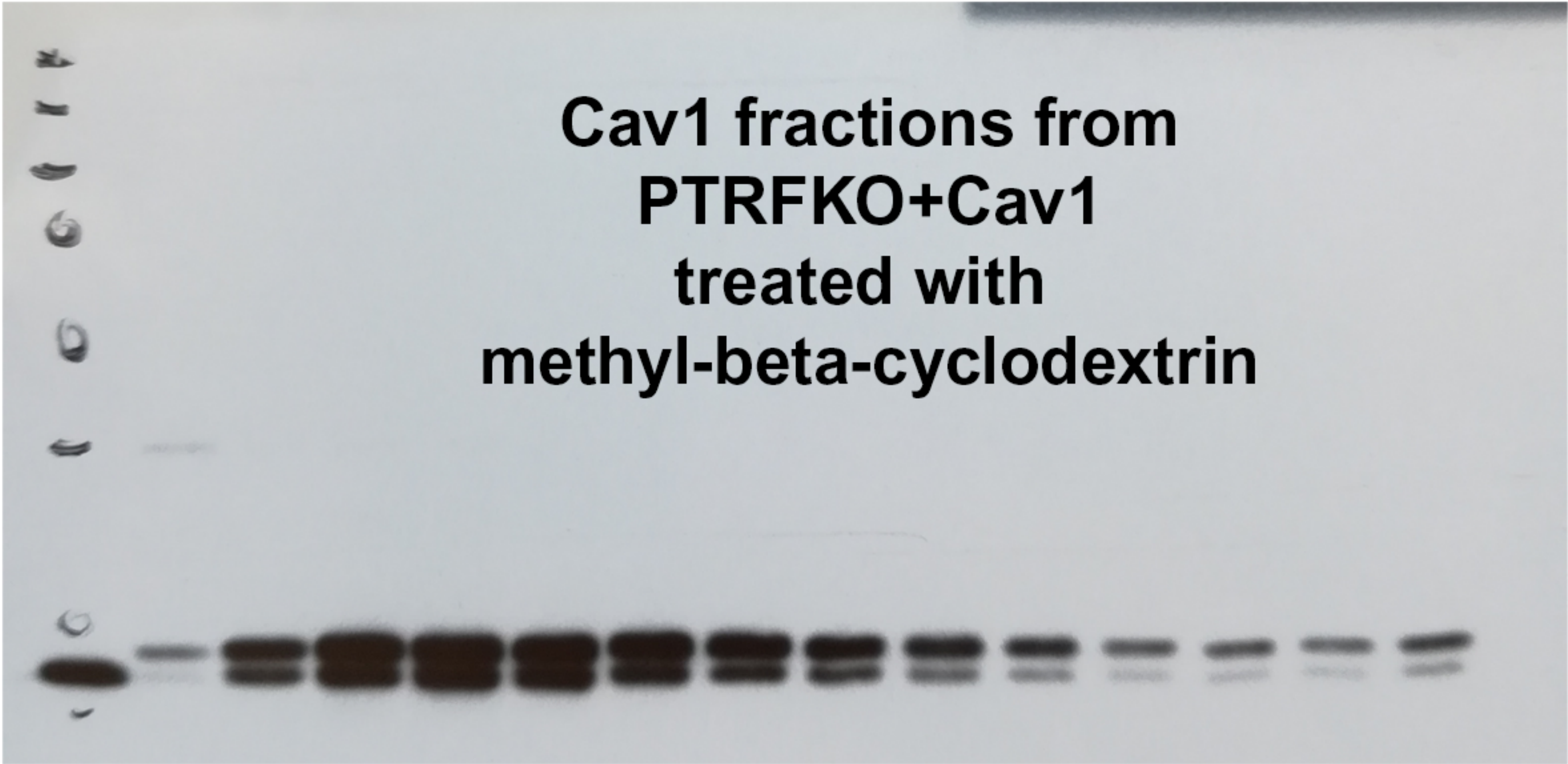

Supplement: Source Data Fig. 7 — Unprocessed western blots from Fig. 7. [file 41556_2022_1034_MOESM25_ESM.pdf]

From Suppl. Figure 1A

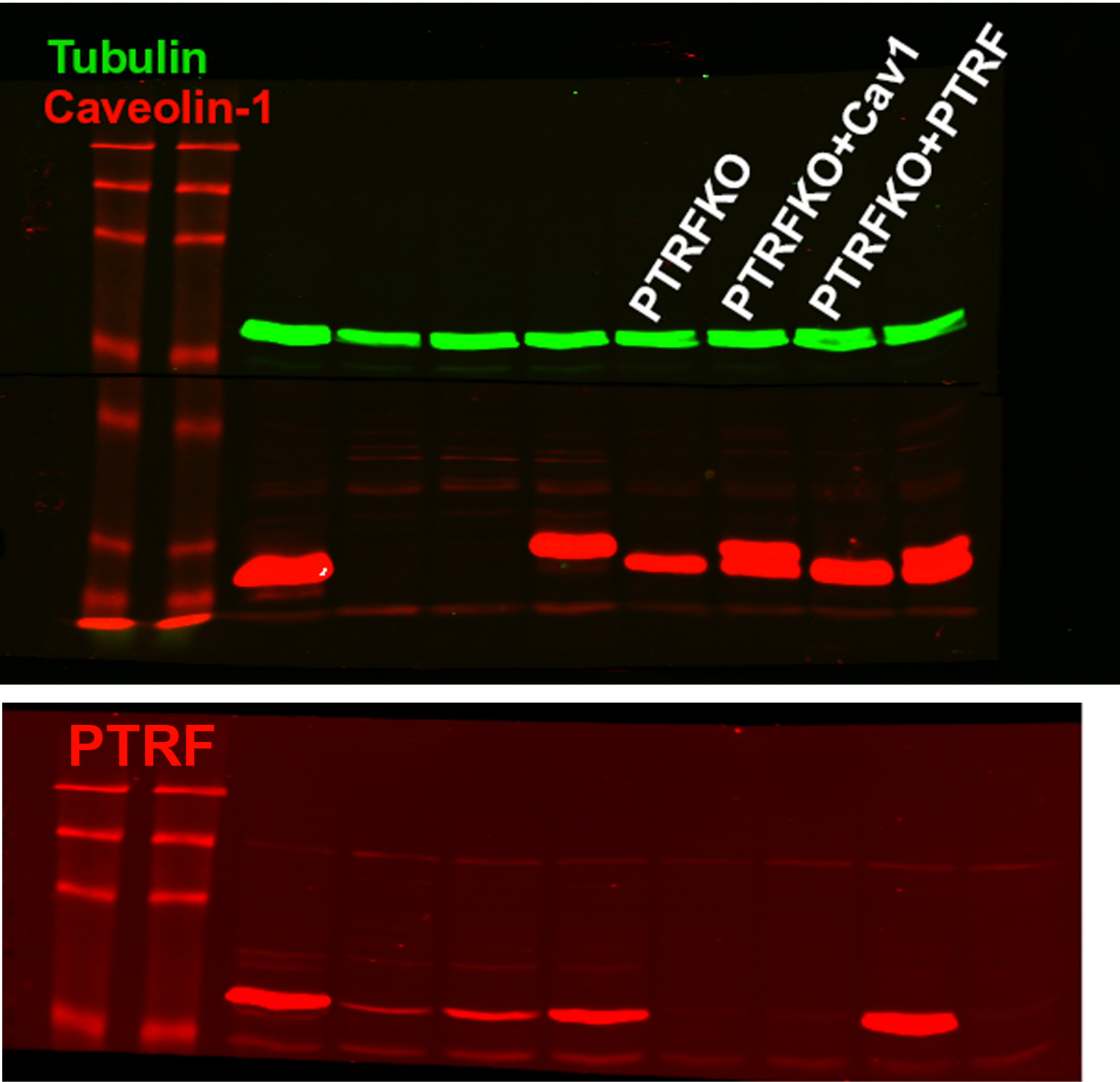

From Suppl. Figure 1G

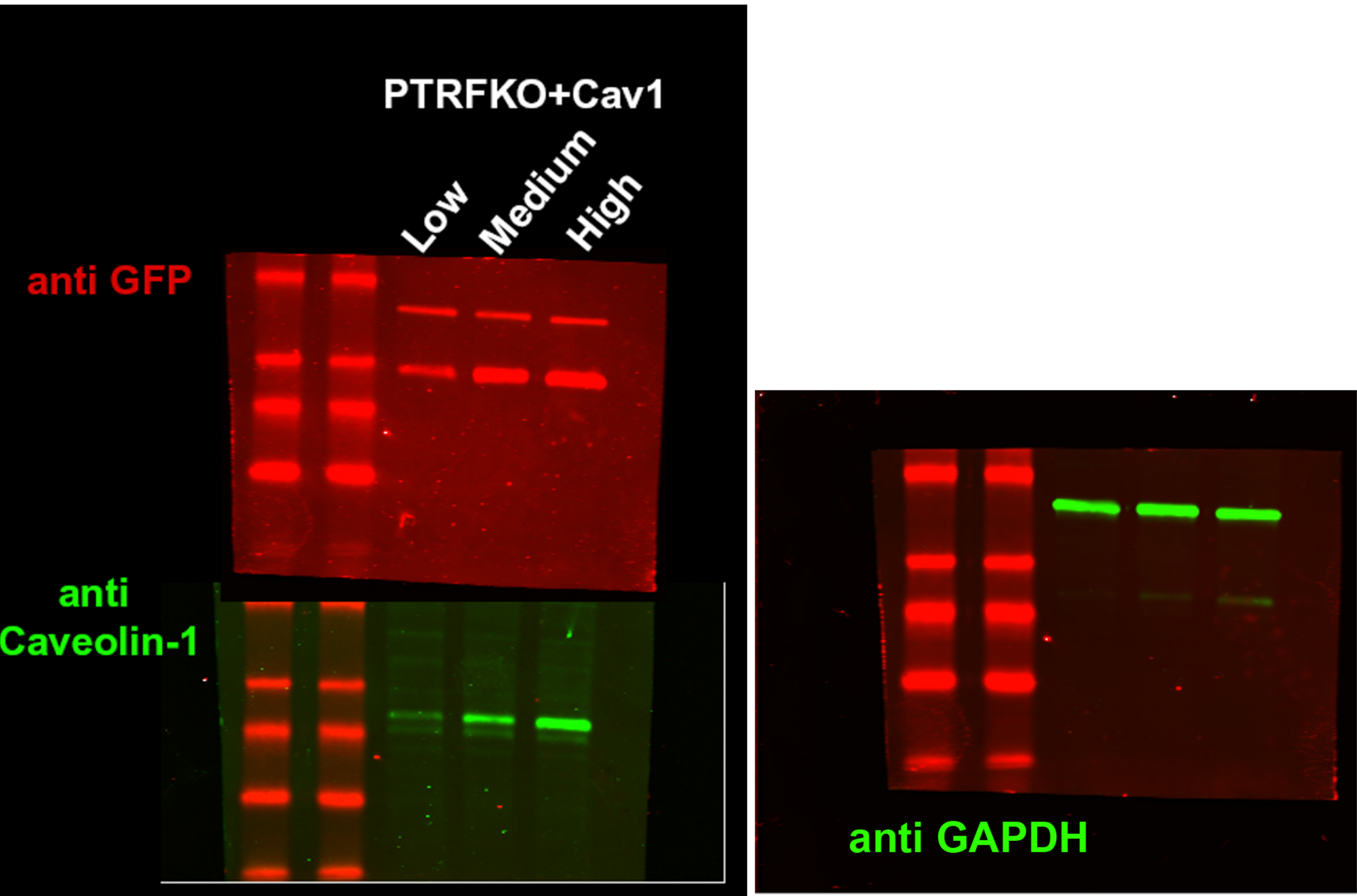

From Suppl. Figure 1B

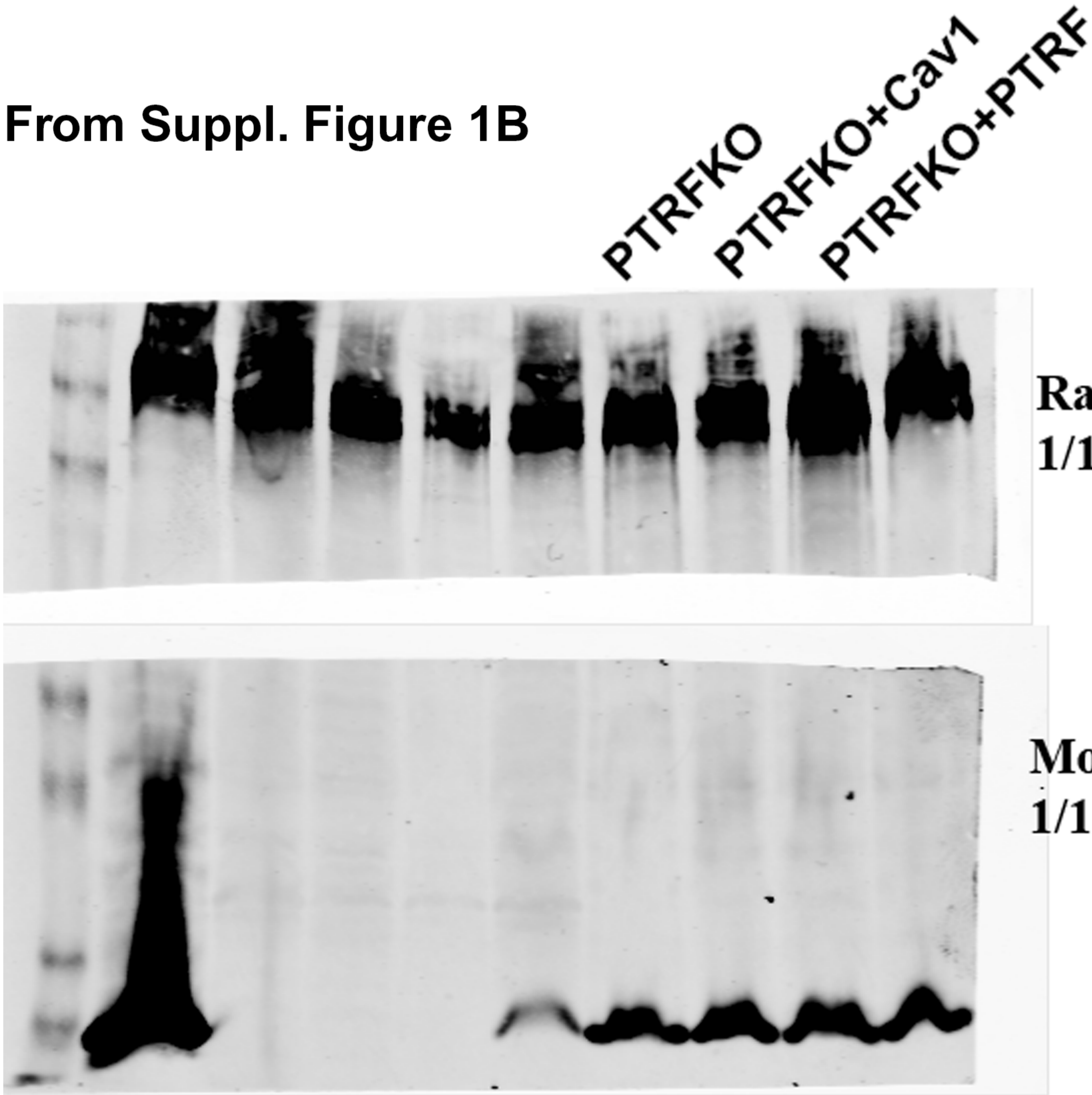

From Suppl. Figure 1E

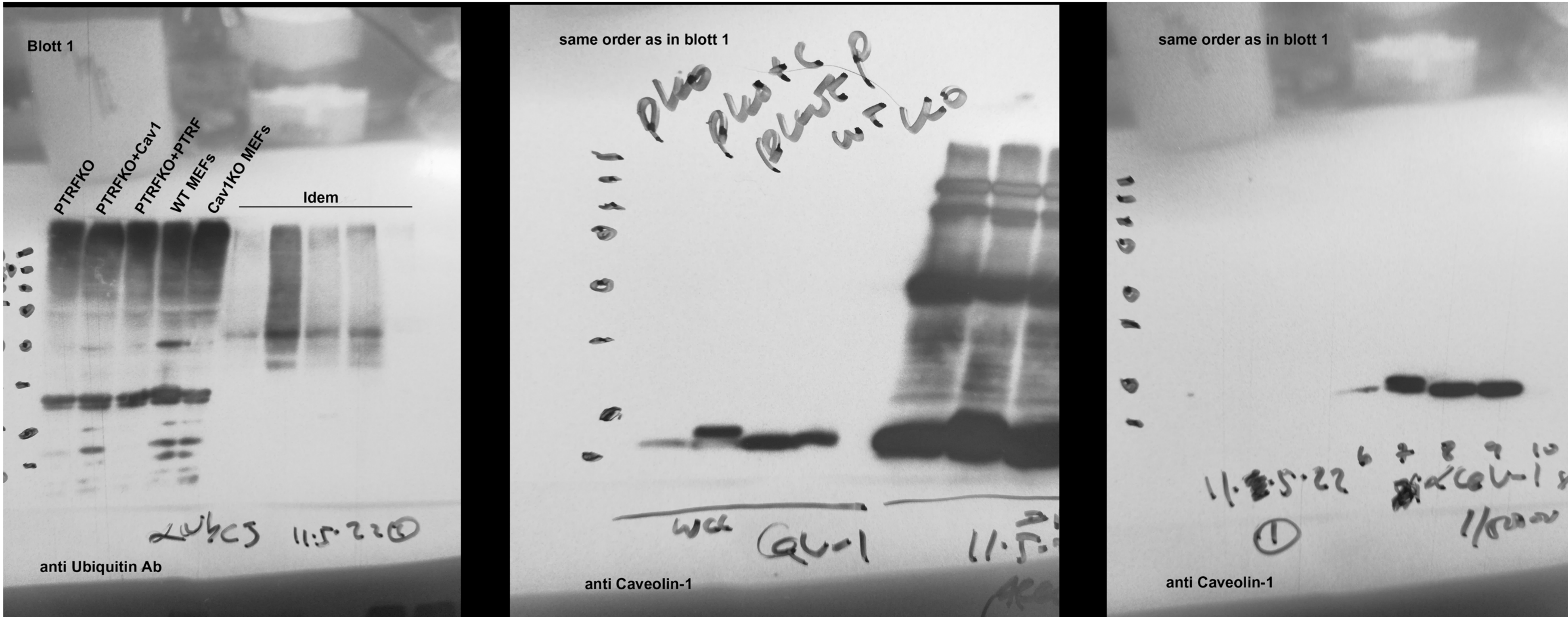

Supplement: Source Data Extended Data Fig. 1 — Unprocessed western blots from Extended Data Fig. 1. [file 41556_2022_1034_MOESM27_ESM.pdf]
